# Sex-Specific Responses to Intermittent Fasting: A Narrative Review Across Physiological, Clinical, and Psychosocial Contexts

**DOI:** 10.3390/nu18101502

**Published:** 2026-05-08

**Authors:** Óscar Fraile-Martínez, Diego Liviu Boaru, Patricia de Castro-Martínez, Miguel A. Ortega, Cielo García-Montero

**Affiliations:** 1 Department of Medicine and Medical Specialities, Faculty of Medicine and Health Sciences, University of Alcala, 28801 Alcala de Henares, Spain; diegoboaru@hotmail.com (D.L.B.); patriciadecastro1999@gmail.com (P.d.C.-M.); miguel.angel.ortega92@gmail.com (M.A.O.); cielo.gmontero@gmail.com (C.G.-M.); 2 Ramón y Cajal Institute of Sanitary Research (IRYCIS), 28034 Madrid, Spain

**Keywords:** intermittent fasting (IF), sex differences, time-restricted eating (TRE), alternate day fasting (ADF), women’s health, men’s health, energy availability, precision nutrition

## Abstract

**Background/Objectives:** Intermittent fasting (IF) has gained increasing attention as a nutritional strategy to improve metabolic health, body composition, and disease-related outcomes. However, its effects are often interpreted as broadly uniform, despite growing evidence that biological sex may modulate fasting responses. This narrative review examines sex-specific differences in the physiological, endocrine, clinical, and psychosocial effects of IF in women and men. **Methods:** We conducted a narrative synthesis of human and preclinical evidence addressing IF protocols, mechanisms, benefits, adverse effects, and sex-related differences. Particular attention was given to substrate metabolism, hormonal regulation, neuroendocrine sensitivity, energy availability, exercise performance, chronic disease management, aging-related outcomes, and psychological or behavioral responses. **Results:** The available literature suggests that women and men share several beneficial responses to IF, including improvements in body composition and cardiometabolic markers, but may differ in the magnitude, tolerability, and mechanistic basis of these effects. Women appear to show greater sensitivity of reproductive and neuroendocrine function to energetic stress, particularly under conditions of low energy availability, high exercise load, or reproductive vulnerability. In contrast, men may exhibit preserved functional outcomes despite measurable endocrine adaptations, including changes in testosterone dynamics. Across both sexes, responses vary according to fasting protocol, nutritional adequacy, baseline metabolic status, life stage, and clinical context. **Conclusions:** Current evidence supports a sex-informed and context-specific interpretation of IF rather than universally applicable fasting prescriptions. Direct sex-comparative studies remain scarce, and many conclusions are inferred from parallel male and female studies. Future research should integrate sex as a core biological variable in precision nutrition and fasting-based interventions.

## 1. Introduction

### 1.1. Background and Objectives

Intermittent fasting (IF) has emerged as one of the most prominent dietary interventions/tools in recent years, garnering substantial attention from both the scientific community and the general public. The prevalence of IF adoption has increased dramatically in recent years, with rates varying across cultures and geographic regions. Recent data recognize that approximately 10% of American adults actively engage in some form of intermittent fasting [1]. In European countries like Spain, the prevalence of IF is of almost 5% [2], in China nearly to 10% [3], approximately 15% in urban Indians, 18–22% in Scandinavian countries and up to 60% in other regions like Saudi Arabia (outside of Ramadan) [1,4].

The rising popularity of IF is driven by accumulating evidence demonstrating its potential benefits for health. IF has been associated with significant improvements in body weight, body mass index (BMI), and cardiometabolic risk factors, including reductions in fat mass, improvements in glucose homeostasis, enhanced insulin sensitivity, and beneficial alterations in lipid profiles [5,6]. Beyond these clinical effects, IF appears to confer benefits through multiple evolutionarily conserved metabolic and cellular mechanisms, including the activation of adaptive stress responses, metabolic switching, enhanced autophagy, improved mitochondrial function, and reduced inflammation [7,8]. Other potential benefits include favorable effects on gut microbiota, pathways related to healthy aging, and improved endothelial and cardiovascular function [9,10,11].

However, the many benefits surrounding IF has also, at times, favored overgeneralized interpretations of its efficacy and safety. Although IF may induce beneficial metabolic and physiological adaptations, it is not a risk-free or universally effective practice, and its effects are likely to depend on the interaction between fasting protocol, nutritional quality, baseline health status, behavioral context, and broader clinical or environmental conditions [12,13]. Accordingly, IF is better understood not as an intrinsically beneficial or harmful intervention, but as a context-dependent exposure whose outcomes may range from adaptive to maladaptive depending on the individual and the circumstances in which it is implemented.

Despite the growing body of research on IF, a critical knowledge gap persists regarding sex-specific responses to these dietary interventions. Emerging evidence suggests that men and women may present different responses to distinct IF protocols, especially in terms of metabolic outcomes and hormonal adaptations [14,15]. These observations support the need for a more sex-aware and context-sensitive interpretation of the literature, particularly given that biological sex interacts with other major determinants of IF response, such as age, reproductive status, metabolic health, physical activity, and disease background.

To address these gaps, the present review first provides a structured overview of IF, including its conceptual basis, main protocols, underlying mechanisms, potential health benefits, associated risks, and the key contextual factors that modulate its physiological and clinical effects. Building on this framework, the subsequent sections focus on sex-specific responses to IF, with particular attention to how these responses manifest across different domains, including exercise performance, chronic disease contexts, aging-related pathways, and psychological and behavioral outcomes. Rather than assuming uniform or directly comparable effects across sexes, this review adopts an integrative perspective, examining how biological sex interacts with broader physiological and environmental determinants to shape IF responses.

### 1.2. Literature Search and Selection Strategy

This narrative review was conducted using a structured, non-systematic approach to identify relevant literature on intermittent fasting (IF), with a particular focus on sex-specific differences in metabolic, neuroendocrine, and clinical responses.

Literature searches were performed using major scientific databases, including PubMed, Scopus, and Web of Science. Search terms included combinations of keywords such as “intermittent fasting”, “time-restricted eating”, “alternate-day fasting”, “sex differences”, “gender differences”, “metabolism”, “hormones”, “exercise”, “aging”, “reproduction”, and “energy availability”. Reference lists of relevant articles were also manually screened to identify additional studies.

Priority was given to peer-reviewed articles published within the last 10–15 years; however, earlier studies were included when considered seminal or necessary for conceptual or mechanistic context. Both human and preclinical (animal and *in vitro or in silico*) studies were considered, particularly in areas where human evidence remains limited, such as aging biology, neuroendocrine regulation, and molecular signaling pathways.

Articles were selected based on their relevance to the thematic structure of the review rather than predefined inclusion or exclusion criteria. Non-peer-reviewed sources were excluded. Given the narrative nature of this review, no formal systematic screening process, quantitative synthesis, or risk-of-bias assessment was performed.

The strength of this approach lies in its flexibility to integrate multidisciplinary evidence and provide a comprehensive conceptual framework. However, it is inherently subject to limitations typical of narrative reviews, including potential selection bias and the absence of formal study appraisal or quantitative meta-analytic methods.

## 2. An Overview of Intermittent Fasting

### 2.1. Concept and Protocols of Intermittent Fasting

IF can be defined as an eating pattern characterized by alternating periods of voluntary fasting and feeding, with variable duration and frequency of food intake restriction [16]. Historically, such fasting–feeding cycles were inherent to human evolution, arising from environmental food scarcity and seasonal variability. These natural fasting episodes were typically synchronized with circadian and seasonal rhythms, contributing to the development of flexible metabolic systems capable of switching between energy substrates [17]. Accordingly, IF may be viewed not as an artificial nutritional innovation, but as a modern re-expression of ancestral metabolic conditions to which human physiology was repeatedly exposed.

With the Neolithic transition, food scarcity became less frequent due to the emergence of agriculture, the formation of villages and cities, and the division of labor. Hence, IF shifted from a ubiquitous physiological stressor to an optional behavioral practice that, paradoxically, challenges the brain’s evolutionary adaptation to seek and consume food. In modern contexts, IF is used both as a therapeutic or lifestyle strategy for the preservation and restoration of health—a practice already recognized by Greek, Roman, and Chinese physicians—and as part of spiritual and religious traditions centered on discipline, observation, and faith [8,18,19,20,21].

From an applied perspective, IF encompasses several dietary protocols that differ in fasting duration, frequency, and practical feasibility, although all are based on repeated cycles of energy restriction and refeeding. One of the most widely adopted approaches is time-restricted eating (TRE) represents one of the most widely adopted IF regimens, involving the confinement of daily food intake to a specific window, typically ranging from 4 to 10 h, with fasting for the remaining hours of each day. Common TRE protocols include the 16:8 method (16 h fasting, 8 h eating window) and more restrictive variants such as the 20:4 approach [22]. TRE interventions can be further subdivided based on the timing of the eating window: early TRE (eating confined to morning and early afternoon hours, typically initiating at 6–8 AM and finishing by 2–4 PM), mid-TRE (eating window in the middle of the day with the last meal between the 5 and 7 PM), late TRE (eating window extending into the evening) or self-selected TRE (chosen by the participant) [23]. From these protocols, early TRE that align food intake with circadian rhythms have demonstrated particularly robust and greater improvements when compared to other TRE protocols in insulin sensitivity, blood pressure, and oxidative stress markers, even in the absence of weight loss [24,25]. Likewise, the duration of the feeding window also appears to modulate the magnitude and nature of these metabolic adaptations. Recent meta-analyses [24] suggest that shorter eating windows (e.g., 4–6 h) may promote greater reductions in body weight and fat mass, albeit at the expense of lean mass preservation, whereas moderate windows (7–9 h) seem more favorable for glycemic control. In contrast, longer windows (10–12 h) may exert more consistent effects on lipid profiles. Collectively, these findings highlight that both the timing and duration of food intake interact to shape metabolic outcomes, reinforcing the need for integrative approaches when evaluating TRE protocols.

Alternate-day fasting (ADF) involves alternating between “feast days” of unrestricted eating and “fast days” with either complete caloric restriction or consumption of approximately 25% of normal energy needs (typically 500–600 calories) [26]. Modified ADF, which permits limited caloric intake on fasting days, has become the predominant approach in clinical trials due to improved adherence and sustainability compared to complete fasting protocols [27]. ADF has consistently demonstrated efficacy in reducing body weight, improving lipid profiles, and enhancing insulin sensitivity [26,28].

Periodic fasting (PF) represents another form of IF distinguished by longer, less frequent fasting periods ranging from 24 h to several consecutive days, followed by refeeding intervals spanning days or weeks [29]. PF protocols may include multi-day fasts (e.g., 2–5 days per month) or fasting-mimicking diets (FMDs) designed to reproduce fasting’s metabolic effects while maintaining minimal caloric intake (usually 30–50% of daily energy needs). This approach has been associated with cellular rejuvenation, enhanced autophagy, and protection against age-related diseases in both animal and human studies [29,30]. The 5:2 diet represents a modified IF approach involving five days of normal eating combined with two non-consecutive days of caloric restriction (approximately 500 calories for women and 600 calories for men) [31]. This protocol offers greater flexibility than daily CR while maintaining metabolic benefits, including improvements in weight loss, glucose homeostasis, and cardiovascular risk factors [32]. Thus, the 5:2 diet can be regarded as a mild, structured form of periodic fasting, while ADF represents a higher-frequency, shorter-duration variant.

Although these protocols differ in structure and intensity, they appear to converge on a shared physiological core: a recurring transition from the fed state to a fasting state that imposes a controlled metabolic challenge and triggers adaptive responses. Various meta-analyses have reported that all major IF models demonstrate potential to improve metabolic health relative to usual dietary patterns, and, as with many lifestyle interventions, long-term adherence and individual fit are likely more relevant than identifying a universally “best” protocol [33]. However, the literature reports that ADF seemed to be slightly superior to other IF strategies for improving metabolic health and weight loss [33,34], although further high-quality trials are warranted to confirm and better contextualize these results.

Beyond these strategies, voluntary fasting has also been consistently embedded in cultural, spiritual, and religious practices across civilizations. Among these, the observance of Ramadan in Islam represents one of the most widespread and explored contemporary models of IF, involving daily abstinence from food and fluid intake from dawn to sunset for approximately one lunar month [35]. Ramadan fasting can be considered as a type of late TRE characterized by a nocturnal feeding pattern, resulting in a marked shift in meal timing, sleep–wake cycles, and circadian alignment [36,37,38]. Importantly, the physiological effects of Ramadan appear to be influenced not only by these factors, but also by cultural dietary patterns, hydration status, and lifestyle changes [38,39,40,41], highlighting the heterogeneity of its effects and the complexity of extrapolating findings from controlled IF interventions to real-world fasting behaviors. In addition to Ramadan, other forms of religious fasting—such as Christian fasting periods, Buddhist fasting practices, Yom Kippur or Hindu observances like Ekadashi—also involve cyclical food restriction patterns that, while heterogeneous in duration and strictness, reinforce the notion that intermittent energy restriction has long been integrated into human behavioral frameworks beyond clinical or nutritional paradigms [35]. These practices further illustrate that the health effects of fasting cannot be fully understood without considering psychosocial, cultural, and behavioral dimensions, which may interact with metabolic responses in ways not captured by standardized experimental protocols [42].

**Figure 1:**
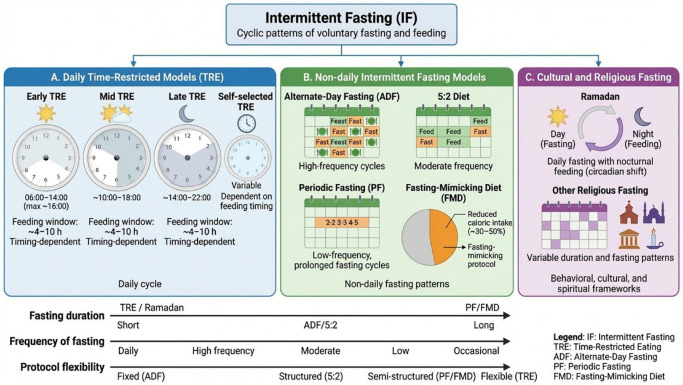
Classification and comparative overview of intermittent fasting (IF) strategies. Intermittent fasting is defined as a pattern of cyclic voluntary fasting and feeding, encompassing multiple protocols that differ in timing, frequency, and duration of food intake restriction. (**A**) Daily time-restricted eating (TRE) models confine food intake to a consistent daily window (~4–10 h) and can be subdivided according to the timing of the feeding period into early, mid, late, and self-selected TRE. These approaches follow a daily cycle and are characterized by timing-dependent effects. (**B**) Non-daily intermittent fasting models include alternate-day fasting (ADF), characterized by alternating fasting and feeding days (high-frequency cycles); the 5:2 diet, involving two non-consecutive days of energy restriction per week (moderate frequency); periodic fasting (PF), consisting of multi-day fasting episodes performed less frequently (low-frequency, prolonged cycles); and fasting-mimicking diets (FMD), which simulate fasting physiology through reduced caloric intake (~30–50%). (**C**) Cultural and religious fasting encompasses structured and unstructured practices embedded in behavioral, cultural, and spiritual contexts. Ramadan represents a form of daily fasting with nocturnal feeding and circadian phase shift, whereas other religious fasting practices are characterized by variable duration and patterns. The lower panel illustrates the relative positioning of these strategies across three key dimensions: fasting duration (short to long), frequency of fasting (daily to occasional), and protocol flexibility (fixed to flexible). Collectively, these models share a common structure of alternating fasting and feeding periods, while differing in their temporal organization and practical implementation. Abbreviations: IF, intermittent fasting; TRE, time-restricted eating; ADF, alternate-day fasting; PF, periodic fasting; FMD, fasting-mimicking diet.

In Figure 1 we summarize the main types and characteristics of IF protocols, understanding that specific considerations and differences may be taken into account for each one.

### 2.2. Health Effects of Intermittent Fasting

Mechanistically, these effects can be interpreted through the biological principle of hormesis. IF acts as a controlled metabolic stressor that elicits adaptive responses when imposed within the organism’s capacity to respond and recover. Hormesis refers to the beneficial effects of low-to-moderate stress exposure, whereas chronic or excessive stress may become detrimental [43]. In this sense, organisms do not enhance adaptive capacity unless challenged to do so; without such a stimulus, biological systems tend to prioritize metabolic economy and maintain only the level of function needed for immediate homeostasis [44]. IF therefore functions as a recurrent hormetic signal (hormetin), cyclically activating metabolic and cellular stress-response pathways rather than maintaining a static adaptive state [45].

A central feature of fasting physiology is the shift from exogenous glucose utilization to endogenous fat mobilization and ketone body production, often referred to as the metabolic switch [19,46]. This transition is orchestrated by reduced insulin and increased glucagon and catecholamines, which stimulate lipolysis and hepatic ketogenesis [19]. Far from being a simple change in fuel source, this switch initiates a coordinated adaptive program that promotes cellular maintenance and repair during fasting and supports growth and plasticity during refeeding [47]. This metabolic flexibility, reflected in metabolomic profiles characterized by elevated ketone bodies, branched-chain amino acids (BCAAs), and markers of enhanced lipid oxidation, likely provided an evolutionary advantage in fluctuating environments characterized by caloric scarcity and low carbohydrate availability [46,48,49]. Among the metabolites generated during fasting, β-hydroxybutyrate (BHB) deserves particular attention because it functions not only as an energy substrate but also as a signaling molecule. BHB has been reported to act as a class I histone deacetylase (HDAC) inhibitor and to suppress the NLRP3 inflammasome, thereby linking fasting metabolism to epigenetic regulation and inflammatory control [50]. This helps explain why IF may exert effects that go beyond those expected from caloric reduction alone.

At the molecular level, IF modulates several conserved nutrient-sensing pathways, including suppression of the mechanistic target of rapamycin (mTOR), activation of AMP-activated protein kinase (AMPK), and upregulation of forkhead box O (FOXO) transcription factors, collectively favoring metabolic regulation, stress resistance, and catabolic efficiency [51,52,53,54]. Additionally, IF enhances sirtuin (SIRT) activity (particularly SIRT1 and SIRT3), which regulates mitochondrial function and cellular stress responses through NAD^+^-dependent deacetylation [55]. In parallel, fasting increases the expression of heat-shock proteins (HSPs), promotes endogenous antioxidant defenses, and improves cellular redox handling [56].

IF also improves mitochondrial efficiency (mitohormesis) through enhanced oxidative phosphorylation and NAD^+^/NADH balance [57]. Additionally, IF may promote mitochondrial biogenesis through PGC-1α signaling and nuclear respiratory factors (NRF1/2), improving mitochondrial density, respiratory capacity, and metabolic flexibility, while reducing reactive oxygen species (ROS) production and inducing the expression of cytoprotective enzymes such as superoxide dismutase (SOD), catalase (CAT), and glutathione peroxidase (GPX), enhancing cellular resistance to oxidative stress [58,59]. Fasting-induced inhibition of IGF-1 signaling has likewise been linked to increased stress resistance and longevity-related pathways through reduced anabolic drive and greater investment in cellular maintenance [60].

Closely related to these effects is the induction of autophagy, which contributes to the removal of damaged proteins and organelles. IF appears to stimulate autophagy through mTOR inhibition and AMPK activation, directly modulating critical orchestrators of this process (i.e., promoting ULK1 and LC3-II activation and preventing p62 accumulation), thereby supporting intracellular quality control and repair [61]. Taken together, these coordinated responses—metabolic switching, mitochondrial optimization, antioxidant upregulation, autophagy, and enhanced stress resistance—can be regarded as hallmark hormetic adaptations linked to improved healthspan and possibly longevity [62].

Beyond host cellular pathways, IF may also influence the gut microbiota and its downstream metabolic and immunological effects. Available studies suggest that IF can increase the richness and abundance of bacterial taxa such as Lactobacillus and Akkermansia, which are often associated with better metabolic health and lower inflammation [63]. IF also appears to regulate the production of critical microbial metabolites such as short-chain fatty acids (SCFAs), which act as signaling metabolites influencing immune modulation and energy homeostasis [64]. However, some studies indicate that the beneficial effects of IF in gut microbiota may return to baseline levels after the fasting periods unless sustained by additional dietary and lifestyle changes [63].

These metabolic and microbial adaptations may also intersect with circadian regulation. Feeding–fasting cycles serve as potent zeitgebers for peripheral clocks and may help synchronize circadian rhythms through pathways involving BMAL1 and CLOCK [65,66,67]. In addition, IF has been associated with reduced inflammatory markers, including C-reactive protein (CRP), tumor necrosis factor alpha (TNF-α), interleukin-1 beta (IL-1β), and IL-6, suggesting that some of its health effects may arise from the convergence of metabolic, circadian, microbial, and immune regulation [68].

**Figure 2:**
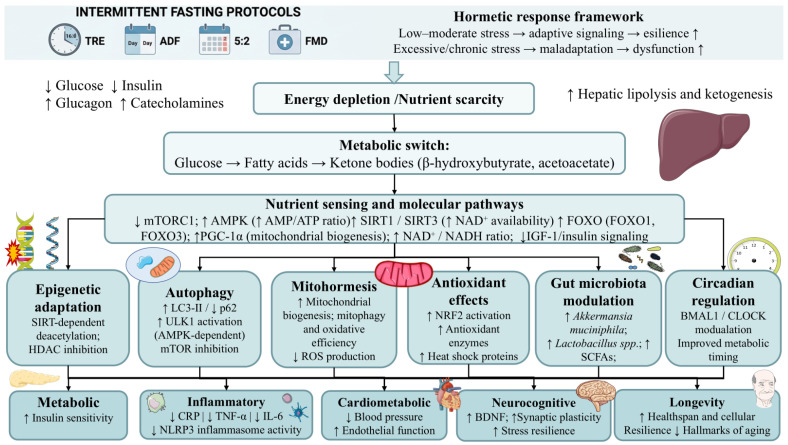
Molecular and physiological framework underlying the adaptive responses to intermittent fasting (IF). IF protocols—including time-restricted eating (TRE), alternate-day fasting (ADF), the 5:2 diet, and fasting-mimicking diets (FMD)—induce a coordinated metabolic response characterized by cycles of energy depletion and nutrient scarcity. These conditions trigger a metabolic switch from glucose utilization to increased fatty acid oxidation and ketone body production (β-hydroxybutyrate and acetoacetate), accompanied by reduced circulating glucose and insulin levels and increased glucagon and catecholamines. At the cellular level, these changes activate key nutrient-sensing pathways, including decreased mTORC1 signaling and increased AMPK activation (reflecting altered AMP/ATP ratios), alongside modulation of sirtuins (SIRT1/SIRT3), FOXO transcription factors, and PGC-1α, collectively promoting mitochondrial biogenesis and metabolic flexibility. These processes are further supported by shifts in redox status (↑ NAD^+^/NADH ratio) and reduced IGF-1/insulin signaling. Downstream, IF elicits a range of adaptive responses, including enhanced autophagy, epigenetic remodeling (e.g., SIRT-dependent deacetylation and HDAC inhibition), mitohormesis, antioxidant defenses (e.g., NRF2 activation), modulation of gut microbiota composition, and improved circadian regulation. These mechanisms operate within a hormetic framework, whereby low-to-moderate metabolic stress promotes adaptive resilience, whereas excessive or chronic stress may lead to maladaptation. Collectively, these molecular and physiological adaptations contribute to improved metabolic health (e.g., enhanced insulin sensitivity), reduced inflammation, improved cardiovascular and neurocognitive function, and increased healthspan and resilience to aging-related processes. Abbreviations: IF, intermittent fasting; TRE, time-restricted eating; ADF, alternate-day fasting; FMD, fasting-mimicking diet; mTORC1, mechanistic target of rapamycin complex 1; AMPK, AMP-activated protein kinase; SIRT, sirtuin; FOXO, forkhead box O; PGC-1α, peroxisome proliferator-activated receptor gamma coactivator 1-alpha; NRF2, nuclear factor erythroid 2-related factor 2; SCFAs, short-chain fatty acids; IGF-1, insulin-like growth factor 1. ↑: increase; ↓: reduction.

Overall, the physiological mechanisms engaged by IF—metabolic switching, mitochondrial remodeling, autophagy, antioxidant defense, circadian synchronization, and inflammatory regulation—appear to reflect evolutionarily conserved programs shaped by intermittent nutrient scarcity, as represented in Figure 2. In this sense, IF may be understood as a contemporary reactivation of ancient adaptive pathways that remain embedded in human biology but are now less frequently engaged under conditions of constant food availability, sedentary behavior, and chronically elevated energy intake [46,49,69]. Nevertheless, despite these multiple mechanistic and health-related benefits, IF is not exempt from risks or limitations, particularly when applied inappropriately or in vulnerable individuals. These considerations are addressed in the following section.

### 2.3. Adverse Effects and Considerations of Intermittent Fasting

Despite the growing body of evidence supporting the metabolic and health-related effects of IF, its implementation is not without potential risks. Rather than being intrinsic to fasting per se, many adverse effects appear to emerge from specific protocol characteristics, individual susceptibility, and contextual factors. This section summarizes the most clinically relevant considerations and evidence-supported risks associated with IF.

#### 2.3.1. Cardiovascular and Metabolic Concerns

Recent epidemiological findings have raised concerns regarding the long-term safety of some IF practices. In particular, an observational analysis of NHANES data [70] suggested a higher cardiovascular mortality risk among individuals reporting extreme TRE (<8 h eating window). However, these findings remain controversial and should be interpreted cautiously, given the observational nature of the study, the possibility of residual confounding and reverse causation, and important limitations in dietary assessment methodology [70]. Even so, they challenge the assumption that fasting is universally safe or inherently cardioprotective across all contexts.

Complementary preclinical data also suggest that prolonged exposure to some fasting regimens may not be benign under all circumstances. Long-term ADF was associated with reduced left ventricular diastolic compliance, increased interstitial myocardial fibrosis, and diminished cardiac reserve in rats [71]. Although extrapolation from animal models to humans must be done cautiously, these findings support the need to consider possible protocol-dependent cardiovascular effects, particularly with prolonged or extreme fasting strategies.

In line with these observations, while short-term IF generally improves lipid profiles, more prolonged ADF may produce lipid abnormalities, particularly elevated LDL cholesterol. In the one-year ADF trial, low-density lipoprotein cholesterol levels significantly increased in the ADF group by 11.5 mg/dL (95% CI, 1.9–21.1 mg/dL) by month 12 compared with the daily CR group [72]. This LDL elevation, occurring despite significant weight loss, represents a concerning and potentially atherogenic consequence of prolonged ADF that may partially explain the increased cardiovascular mortality observed in certain populations. Although these studies do not establish definitive causality and acknowledge potential confounding factors, the findings sufficiently challenge the prevailing assumption that fasting represents a risk-free approach [73].

#### 2.3.2. Gastrointestinal, Gallbladder and Glycemic Effects

Fasting has been clinically associated with gallbladder sludge and pigment gallstone development through mechanisms involving increased biliary calcium, bilirubin, and cholesterol saturation. Short-term fasting (particularly 15–20 h) increases gallbladder bile calcium concentration, total bilirubin, and biliary cholesterol saturation index while decreasing biliary pH, all changes promoting stone formation [74].

Dietary composition substantially influences gallstone risk: Individuals consuming high-carbohydrate meals before fasting display greater stone formation risk, whereas those consuming higher-fat, lower-carbohydrate diets while maintaining a regular diet and avoiding random and frequent eating tend to have reduced risk [75]. Therefore, IF may not be favorable for people susceptible or prone to suffer from gallbladder sludge.

Another relevant safety issue concerns hypoglycemia in individuals with diabetes who are treated with insulin or other glucose-lowering medications. Although IF may improve glycemic control in insulin-resistant states, fasting can increase the risk of clinically significant hypoglycemia if medication doses are not appropriately adjusted at the onset of the intervention [76]. For this reason, IF in medicated patients with diabetes should only be undertaken with appropriate medical supervision and individualized therapeutic adjustment.

#### 2.3.3. Nutritional Deficiencies and Lean Mass Loss

Another critical limitation of IF involves the substantially elevated risk of micronutrient deficiencies when dietary quality is not carefully prioritized. Deficiencies are primarily driven by lower total energy intake and reduced consumption of nutrient-dense foods during fasting periods or within restricted eating windows. Randomized controlled trials implementing ADF and TRE have reported reductions in intake of calcium, magnesium, potassium, folate, vitamin C, and vitamins from the B complex [77].

A related concern is the potential loss of fat-free mass. When IF results in an unintended caloric deficit and insufficient protein and carbohydrate intake—particularly in lean individuals, athletes, or those with limited nutritional knowledge—it may reduce skeletal muscle mass, muscle strength, and resting metabolic rate [78,79,80]. By contrast, IF protocols that are adequately planned and that include sufficient protein intake, appropriate total energy intake, and resistance training may preserve lean mass comparably to—or in some cases better than—continuous calorie restriction [81,82]. Thus, muscle loss is not an inevitable consequence of IF, but rather a risk that emerges when fasting is poorly designed or combined with unintended underfeeding.

This issue is particularly relevant in lean individuals, athletes, older adults, and individuals with limited nutritional knowledge, in whom an excessive fasting “dose” may compromise both performance and long-term metabolic health. Overall, these observations underscore the importance of nutritional education and, where appropriate, supervision by qualified health professionals when IF is prescribed or self-implemented.

#### 2.3.4. Psychological and Behavioral Risks

A clinically relevant and often underappreciated concern is the association between IF and eating disorders (ED) behaviors. Cross-sectional evidence suggests that IF is commonly practiced among adolescents and young adults and is associated with ED psychopathology across sexes, with particularly strong associations reported in females and in transgender or gender non-conforming individuals [83,84]. However, causality cannot be inferred from these data, and it remains unclear whether IF contributes to the development of ED, is preferentially adopted by individuals already at risk, or both [83].

More recent observational and prospective evidence further supports this association, indicating that self-initiated fasting practices may be linked to disordered eating patterns, including binge eating, orthorexia, and increased ED-related symptomatology over time, particularly in younger populations and in those with pre-existing vulnerability [85,86]. These findings, largely derived from real-world and behavioral studies, suggest that fasting behaviors may act as a potential risk marker—or in some contexts a contributing factor—for maladaptive eating patterns, although the directionality of this relationship remains incompletely understood. Importantly, emerging data suggest that this risk is highly context-dependent, with differences observed between structured, supervised interventions and unsupervised, self-directed fasting behaviors [83,85,87].

For this reason, the relationship between IF and ED deserves careful consideration, particularly in women, and will be addressed in greater detail in subsequent sections, where sex-specific and context-dependent psychological responses are examined in depth (see Section 4).

#### 2.3.5. Adherence and Tolerability

From a practical perspective, one of the main limitations of IF is the difficulty some individuals experience in sustaining it over time, particularly with more restrictive regimens such as ADF. In a one-year randomized trial comparing ADF with daily CR in adults with obesity, dropout rates were higher in the ADF group than in the CR and control groups, and dissatisfaction with the regimen was a more frequent reason for withdrawal [28]. These findings suggest that, despite its theoretical simplicity, IF may be difficult to maintain for some individuals in real-world settings.

In parallel, IF can have some associated adverse effects that may impair the adherence to this intervention, particularly at initial stages. The most commonly reported short-term adverse effects of IF include excessive hunger, headaches, dizziness, gastrointestinal disturbances (indigestion, diarrhea, nausea, bloating), irritability, fatigue, ketotic breath, sleep disturbances, and dehydration [88]. While these effects typically diminish during the adaptation phase, they contribute to intervention non-compliance and represent a symptomatic burden that should not be minimized when counseling patients.

#### 2.3.6. Populations Requiring Caution

IF may not be appropriate for all individuals. Caution is warranted—or medical supervision is required—in pregnant or lactating women [89], children and adolescents [90], frail older adults [91], individuals with current or past eating disorders [87], patients with insulin-treated or poorly controlled diabetes, and those with complex chronic conditions [92]. Particular caution is also warranted in individuals taking medications that require food intake for safety, absorption, or pharmacodynamic stability.

Taken together, these considerations indicate that the safety and efficacy of IF cannot be evaluated independently of individual and contextual factors. Rather, the balance between potential benefits and risks depends on the interaction between fasting characteristics, nutritional adequacy, physiological status, and behavioral context, among others. Therefore, the main determinants of the effectiveness of IF will be subsequently discussed.

### 2.4. Critical Factors Influencing Intermittent Fasting Responses

IF, as outlined in previous sections, has been associated with a range of metabolic and physiological effects, as well as potential adverse outcomes in specific contexts. A critical point, therefore, is that IF should not be interpreted as an intrinsically beneficial or harmful intervention, but rather as an exposure whose effects depend on the interaction of multiple biological, nutritional, behavioral, and clinical determinants. Indeed, evidence from randomized controlled trials and meta-analyses indicates that IF, on average, induces weight loss and improves several intermediate cardiometabolic outcomes, often to a similar extent as continuous energy restriction, while between-study variability remains substantial and long-term effects are less well established [93].

This variability can be better understood when IF is framed within a systems biology perspective. Humans are complex adaptive systems in which metabolic phenotypes arise from dynamic interactions across endocrine, neural, immune, and behavioral networks, rather than from isolated inputs [94,95,96]. In this context, IF does not act as a singular causal driver, but as a physiological “signal” that is interpreted and modulated across multiple regulatory layers, including molecular pathways, organ crosstalk, circadian rhythms, and environmental constraints [13,97,98,99]. Consequently, similar fasting protocols may produce markedly different outcomes depending on the individual and situational context in which they are implemented.

Within this framework, IF can be conceptualized as a physiological stressor capable of eliciting adaptive or maladaptive responses depending on the organism’s capacity to respond and recover. Under appropriate conditions—adequate nutrient intake, sufficient recovery, and manageable external stress—fasting may promote beneficial adaptations in metabolic flexibility, insulin sensitivity, and cellular stress resistance. However, when superimposed on conditions of insufficient energy availability, poor sleep, high psychosocial stress, or underlying disease burden, the same fasting stimulus may shift toward maladaptive responses. The concept of allostatic load, defined as the cumulative physiological burden imposed by repeated or chronic stress activation [100], provides a useful bridge between these mechanistic insights and real-world variability.

Importantly, this perspective aligns with the broader concept of inter-individual variability, which is increasingly recognized as a central feature of nutritional science rather than a source of statistical noise [101]. Evidence from precision nutrition demonstrates that individuals can exhibit highly divergent metabolic responses to similar dietary exposures, supporting the notion that “the same intervention may lead to different phenotypes” [102,103]. This principle is clearly reflected within IF research. For instance, while some TRE trials report modest improvements in weight loss and cardiometabolic markers, others show minimal or no additional benefit compared to standard eating patterns [104]. Such discrepancies are unlikely to reflect a simple contradiction in efficacy, but rather differences in contextual factors such as timing of food intake, baseline metabolic status, dietary composition, adherence, and co-interventions.

From a mechanistic and applied perspective, these differences can be understood through several interacting domains of determinants. First, the physiological and biological context plays a foundational role, with baseline metabolic health, circadian biology, hormonal milieu, and genetic and epigenetic factors influencing both the magnitude and mechanisms of response [13,105,106,107]. Within this domain, biological sex and reproductive status should be considered key modifiers, given their influence on substrate utilization, endocrine responses, and energy regulation [14] although these aspects will be examined in detail in subsequent sections.

Second, the nutritional context is a major determinant of IF outcomes. In many studies, observed benefits appear largely mediated by sustained caloric deficit and weight loss rather than fasting per se, with comparable outcomes often reported between IF and continuous energy restriction when energy intake is matched [5,104,108,109]. In addition, protein intake and distribution are critical for lean mass preservation, while macronutrient composition and overall dietary quality influence glycemic control, satiety, and metabolic flexibility [80,110]. Additionally, compressed eating windows may compromise micronutrient intake if dietary diversity is not actively maintained, an aspect that remains insufficiently addressed in many IF studies [77].

Third, the behavioral and lifestyle context strongly modulates both the efficacy and tolerability of IF. Physical activity, particularly resistance training, can mitigate lean mass loss during energy restriction, whereas endurance training interacts with substrate utilization and perceived exertion [82,111]. However, many studies fail to adequately control for training variables, limiting interpretability. Sleep quality and circadian alignment are particularly important, as sleep restriction and circadian misalignment are independently associated with impaired metabolic regulation [112,113] and may attenuate the benefits of IF. Furthermore, psychosocial stress and overall lifestyle burden can influence appetite regulation, adherence, and endocrine responses to IF [114,115], reinforcing the notion that IF cannot be evaluated in isolation from the broader behavioral environment.

Finally, the clinical and environmental context determines both safety and real-world applicability. The presence of chronic disease, medication use, and baseline functional status can substantially alter both the risks and benefits of IF [116,117]. For example, in individuals with insulin-treated diabetes, fasting may increase the risk of hypoglycemia without appropriate monitoring and adjustment [118]. At the same time, structured and supervised protocols may be feasible and beneficial in selected populations, highlighting the importance of individualized implementation. Environmental and sociocultural factors—including work schedules, food availability, socioeconomic status or religious practices—further shape adherence and response to IF and other health determinants [2,104,119], illustrating how context can override the physiological effects of fasting alone.

**Figure 3:**
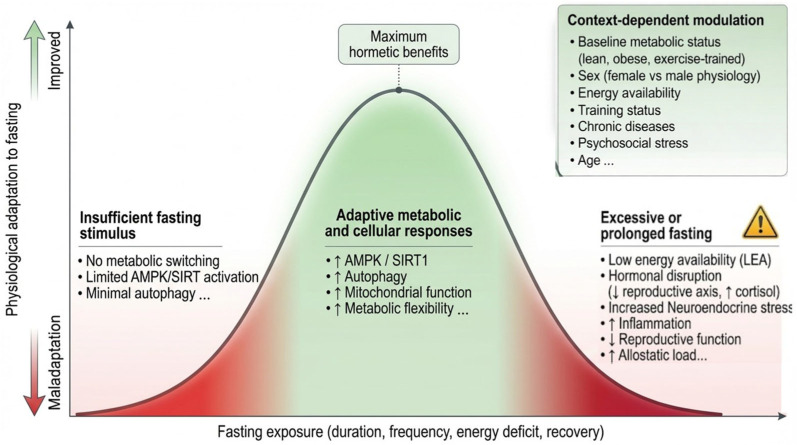
Hormetic response to intermittent fasting exposure. The physiological effects of intermittent fasting follow a hormetic dose–response curve. Insufficient fasting stimulus results in limited metabolic switching and minimal activation of adaptive pathways. In contrast, moderate fasting exposure promotes optimal metabolic and cellular adaptations, including activation of AMPK/SIRT1 signaling, autophagy, improved mitochondrial function, and enhanced metabolic flexibility. Excessive or prolonged fasting may lead to maladaptive responses, such as low energy availability, hormonal disruption, increased stress and inflammation, and impaired physiological function. The magnitude and direction of these responses are modulated by individual factors, including baseline metabolic status, sex, energy availability, training status, chronic disease, psychosocial stress, and age. ↑: increase; ↓: reduction.

Overall, IF should be conceptualized not as an intrinsically beneficial or harmful intervention, but as a context-sensitive exposure whose effects emerge from the interaction of multiple determinants (Figure 3). Differences in outcomes across studies are therefore more likely to reflect variation in these factors than inherent inconsistencies in efficacy. Within this multidimensional model, biological sex represents a major interacting determinant, intersecting with substrate metabolism, endocrine regulation, and recovery capacity. Accordingly, the following sections will examine sex-specific responses to IF while maintaining the systems-level perspective established here.

## 3. Sex-Specific Differences in Response to Intermittent Fasting

Biological responses to IF differ between women and men as a result of complex interactions among sex hormones, substrate metabolism, body composition, and neuroendocrine regulation. Available evidence suggests that these differences are not merely quantitative, but may also affect the direction, magnitude, and tolerability of fasting-induced adaptations [120]. Importantly, both “sex” (biological attributes) and “gender” (sociocultural context) may shape observed responses to IF—sex through endocrine and metabolic regulation, and gender through behavioral patterns, adherence constraints, and symptom reporting—thus, sex-stratified analyses should ideally be interpreted alongside gender-related determinants of exposure [121].

During short-term fasting (14–22 h), women exhibit greater reliance on lipid metabolism while men demonstrate preferential carbohydrate utilization [14]. After 38 h of fasting, plasma glucose concentrations are significantly lower in women compared to men, while free fatty acids (FFAs) and lipolytic rates are markedly higher in women [122]. These sexually dimorphic metabolic patterns become more pronounced during prolonged fasting (38–72 h), with women showing sustained elevations in plasma glycerol and FFA levels [14]. Experimental data further suggest that women are relatively protected from FFA-induced insulin resistance during fasting, possibly by limiting intramyocellular ceramide accumulation despite higher circulating FFA, whereas men show a tighter coupling between lipid oversupply and insulin resistance [15,122].

However, the mechanistic basis for this protection should be framed cautiously. For instance, after prolonged fasting (~38 h), muscle ceramide content did not differ significantly between sexes and did not correlate with peripheral glucose uptake, indicating that ceramide accumulation should be considered a plausible—but not definitively established—mediator in humans [122]. In addition, fasting may redistribute lipid to different ectopic depots in a sex-specific manner. During a 48 h fast, liver triglyceride content increased in men (especially from ~4 to 24 h), whereas intramyocellular triglyceride increased in women (not men) from ~24 to 48 h, highlighting that lipid handling differences extend beyond circulating FFA to tissue partitioning with potential metabolic implications upon refeeding [123]. Consistent with a tissue-partitioning framework, some data indicate that under extended overnight/prolonged postabsorptive conditions, young women may channel circulating FFA more toward ketone body production relative to very low-density lipoprotein (VLDL)-triacylglycerol synthesis than young men, implying potentially faster or more pronounced metabolic switching under certain conditions [124]. Nevertheless, ketone responses are not consistently higher in women across all fasting paradigms, as some studies report no significant sex differences in circulating ketone concentrations after prolonged fasting (~48 h), suggesting that ketogenesis is modulated by additional variables such as fasting duration, adiposity, and hormonal milieu [123]. Furthermore, exogenous hormonal modulation—such as hormonal contraception—may alter substrate utilization during fasting, with evidence indicating reduced glucose oxidation after ~22 h of fasting in women using oral contraceptives compared to naturally cycling women [125].

These metabolic differences likely reflect distinct evolutionary pressures and body-composition constraints, whereby females evolved enhanced capacity for fat storage and mobilization to support gestation and lactation, and males evolved greater reliance on carbohydrate oxidation to sustain acute physical effort [126]. Given the inherently inferential nature of evolutionary explanations, this framework is most robust when grounded in well-characterized sex differences in adipose tissue distribution and fatty acid trafficking—such as higher total adiposity and preferential subcutaneous/gluteofemoral fat deposition in women versus greater abdominal/visceral fat accumulation in men, alongside sex-specific differences in FFA uptake, re-esterification, and lipolysis—which plausibly align with reproductive energetic demands [127]. Although evolutionary interpretations should be made cautiously, this framework is broadly consistent with experimental and comparative animal data showing sex-dependent responses to IF, including differential effects on longevity and reproduction according to the fasting protocol employed [128]. Such findings support the notion that the biologically “optimal” fasting dose may not be the same in females and males.

Hormonal regulation represents another critical dimension of sex differences in IF responses. Women are often described as having higher kisspeptin activity, a neuropeptide essential for reproductive function that is highly sensitive to changes in energy availability [129,130]. However, a more precise characterization is that the kisspeptin system is sexually dimorphic in a region- and steroid-dependent manner (e.g., greater kisspeptin neuron density and activity in the anteroventral periventricular nucleus in females, with more comparable expression in the arcuate nucleus), and human data similarly support sex differences in hypothalamic kisspeptin signaling; thus, it may be more accurate to refer to “sexually dimorphic kisspeptin circuitry” rather than a uniform elevation in women [131]. Fasting-induced reductions in kisspeptin can disrupt gonadotropin-releasing hormone (GnRH) pulsatility, potentially affecting the entire hypothalamic–pituitary–gonadal (HPG) axis [132]. In rodent models, even relatively short fasting or intermittent restriction reduces hypothalamic KiSS1 expression in estrogen-primed females, attenuates luteinizing hormone (LH) pulses, and advances or disrupts estrous cyclicity, underscoring the susceptibility of the female reproductive axis to negative energy balance when estrogen is present [133,134]. In humans, short-term fasting (3 days) during the mid-follicular phase has also been shown to reduce LH pulse frequency and amplitude, although ovulation may still occur in healthy, normally cycling women [135,136], suggesting that transient neuroendocrine perturbations do not necessarily translate into overt reproductive dysfunction in energy-replete individuals.

Beyond kisspeptin–GnRH coupling, other energy-sensing signals interacting with reproductive and metabolic regulation also display sex-specific characteristics. Leptin—primarily secreted by white adipose tissue and acting as a key regulator of energy balance and satiety—is consistently higher in women than in men, even after adjustment for fat mass, suggesting that factors beyond total adiposity contribute to this sexual dimorphism [137,138]. Fasting induces a marked reduction in circulating leptin levels in both sexes; however, available evidence suggests that women may experience a more pronounced relative decline in leptin during prolonged fasting, effectively abolishing the baseline sex difference observed in the fed state [139]. This greater leptin suppression, together with concomitant increases in FFAs and reductions in insulin and glucose, may amplify central signals of energy deficit and contribute to tighter neuroendocrine coupling between energy availability and reproductive function in women.

Beyond the HPG axis, sex differences in stress and energy-sensing pathways may further shape responses to IF. Kisspeptin neurons integrate metabolic and glucocorticoid signals, providing a mechanistic point of convergence between nutritional status, stress physiology, and reproductive output. Experimental evidence in murine models indicates that fasting or stress acting through glucocorticoid-sensitive pathways may simultaneously alter energy metabolism and suppress gonadotropin secretion [140,141]. In parallel, women appear to show distinct counterregulatory and sympathetic responses to fasting and hypoglycemia, which may influence both fasting tolerability and downstream metabolic outcomes [14]. Together, these mechanisms suggest that women’s reproductive and neuroendocrine systems may be more tightly coupled to energy status than those of men, especially under conditions of repeated fasting, high training load, LEA, or other concurrent stressors. Importantly, available evidence suggests that many of the adverse reproductive and neuroendocrine effects described in women are primarily driven by sustained LEA rather than fasting per se, particularly when fasting protocols result in insufficient total caloric intake relative to physiological demand [84,142]. A practical implication for interpreting sex comparisons is that “the same clock-time fast” can correspond to different relative energetic strain because of sex differences in lean mass and baseline energy expenditure [143]. Thus, sex contrasts are most interpretable when fasting exposure is contextualized by energy balance/availability and protein distribution, not only fasting-window duration [144,145].

At the clinical level, these mechanistic differences have important implications. Emerging evidence suggests that men and women may require different IF “doses” in terms of fasting duration, timing, and frequency to achieve similar benefits while minimizing adverse effects [146,147,148]. However, interpretation remains limited by the fact that many IF trials either underrepresent women or fail to stratify outcomes by sex. Here it may be worth clarifying that representation biases can run in both directions depending on study type: mechanistic laboratory fasting physiology has historically under-enrolled women (often citing cycle-related variability), whereas behavioral weight-loss interventions—including many diet trials—often under-enroll men; both patterns reduce the resolution of sex-specific inference for IF prescriptions [144]. Moreover, not all clinically relevant endpoints show strong sex divergence. In secondary analyses pooling short-term ADF cohorts in adults with obesity, weight loss and improvements in insulin resistance did not generally differ by sex or menopausal status (with some lipid endpoints showing group-specific differences) [149], illustrating that sex effects may be endpoint- and protocol-dependent rather than universal.

Even so, the available literature points to sex-specific differences in body composition responses, lipid handling, glycemic variability, and adverse outcomes such as menstrual disturbances or changes in androgen-related parameters [84,120,148]. Mathematical and systems biology models further support the view that sex-specific differences in hepatic metabolism, adipose tissue dynamics, glycogen handling, and hormonal feedback loops can produce divergent metabolic trajectories under the same fasting regimen [150]. Importantly, recent controlled analyses indicate that when pretesting conditions are standardized (e.g., diet and physical activity for ≥24 h) and menstrual cycle phase is accounted for, women do not exhibit greater within-individual variability than men in key fasting metabolic parameters—including plasma glucose, insulin, lactate, triglycerides, FFAs, resting metabolic rate, and body mass—despite the expected higher variability in circulating sex hormones [144]. This challenges the longstanding assumption that female hormonal cyclicity introduces excessive noise in metabolic research and supports the feasibility of including women without compromising statistical power or increasing resource requirements, although the influence of menstrual cycle phase should not be dismissed as phase-dependent fluctuations in estrogen and progesterone have a significant impact on nutrient metabolism, along with other extra-hormonal mechanisms associated with menstrual cycle [151]. Interestingly, some data also support the “greater male variability” hypothesis in human metabolism [144,152] with men displaying higher between-individual variability in resting metabolic rate—likely reflecting greater heterogeneity in fat-free mass and physical activity patterns—further challenging the assumption that females inherently introduce greater variability in metabolic research.

**Figure 4:**
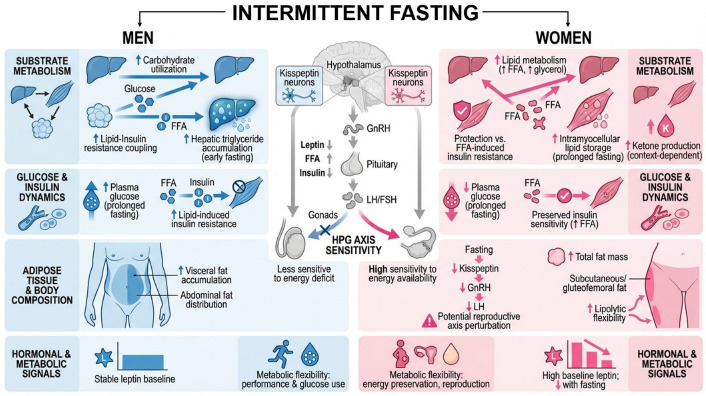
Sex-specific differences in metabolic and neuroendocrine responses to intermittent fasting. Intermittent fasting elicits distinct metabolic and hormonal responses in men and women. In men, fasting is generally associated with greater reliance on carbohydrate utilization, increased lipid-induced insulin resistance, and preferential visceral fat accumulation, alongside relatively stable leptin levels and lower sensitivity of the hypothalamic–pituitary–gonadal (HPG) axis to energy deficit. In women, fasting promotes enhanced lipid metabolism and lipolytic flexibility, preservation of insulin sensitivity despite elevated free fatty acids, and a higher sensitivity of the HPG axis to changes in energy availability. This increased sensitivity may contribute to alterations in reproductive hormone signaling (e.g., kisspeptin–GnRH–LH axis) under conditions of energy deficit. Collectively, these differences highlight the importance of considering sex-specific physiology when evaluating the metabolic and endocrine effects of intermittent fasting. ↑: increase; ↓: reduction.

Collectively, these findings indicate that biological sex is not a minor covariate, but a major determinant of metabolic, neuroendocrine, and clinical responses to IF. The main differences and similarities between women and men’s response to IF are summarized in Figure 4. This supports the need for a sex-informed interpretation of the literature and argues against fully sex-neutral fasting prescriptions. The following sections therefore examine the specific effects, risks, and clinical considerations of IF in women and men separately.

### 3.1. Specific Considerations of Intermittent Fasting in Women

The effects of IF in women should be interpreted within the context of a highly energy-sensitive reproductive and neuroendocrine system. In contrast to men, female reproductive physiology is tightly coupled to energy availability, metabolic status, and stress signaling. Consequently, menstrual status, life stage, body composition, exercise load can all substantially modulate the balance between the potential benefits and potential risks of fasting interventions.

#### 3.1.1. Women of Reproductive Age

##### Neuroendocrine and Menstrual Responses to Intermittent Fasting

Biological responses to IF in reproductive-age women are strongly influenced by the dynamic interplay between energy availability, neuroendocrine regulation, and cyclical changes in substrate metabolism across the menstrual cycle. Beyond the well-established sensitivity of the HPG axis to energetic stress, physiological fluctuations in ovarian hormones also modulate nutrient partitioning, substrate utilization, and appetite regulation, thereby shaping how fasting stimuli are perceived and tolerated across the cycle [151]. Controlled studies outside the IF literature show that estradiol peaks in the late follicular phase are associated with lower spontaneous energy intake and greater reliance on carbohydrate oxidation, whereas the progesterone-dominated mid-luteal phase is characterized by increased energy intake, higher hunger, and a relative shift toward greater lipid and protein utilization [151,153]. These cyclical changes suggest that the metabolic “baseline” upon which fasting is imposed is not static in women, but varies meaningfully across the menstrual cycle. As a result, identical fasting protocols may impose different relative energetic stress depending on cycle phase, with potential implications for both adherence and physiological outcomes.

Evidence from controlled human studies suggests that short-term fasting can modify reproductive hormone dynamics in women without necessarily impairing ovulatory function. In normal-weight, normally cycling women, a 3-day fast during the mid-follicular phase reduced LH pulse frequency and blunted the expected increase in mean LH levels and pulse amplitude across the early-to-mid-follicular window, yet follicular development, ovulation, and overall cycle length were preserved [136]. By contrast, in lean women with low body fat, a comparable 72 h fast induced more pronounced neuroendocrine alterations, including higher evening cortisol, suppression of the nocturnal TSH rise, lower T3 concentrations, and a reduction in LH pulsatility, together with evidence of greater reproductive disruption: some cycles became anovulatory and follicular phase length was prolonged in most women studied under both fed and fasted conditions [154]. These findings suggest that acute fasting may be tolerated with relative short-term reproductive resilience in healthy, energy-replete women, but that women with lower energy reserves may be more susceptible to fasting-induced neuroendocrine perturbation and impaired ovulatory dynamics. In parallel, recent reviews of intermittent fasting interventions in humans suggest that, in premenopausal women with obesity, fasting regimens—particularly TRE protocols—may reduce androgen-related markers such as testosterone and the free androgen index while increasing sex hormone-binding globulin (SHBG), with little evidence of consistent effects on estrogens, gonadotropins, or prolactin of IF in women [148].

Real-world fasting models provide complementary insight into how these mechanisms operate under less controlled conditions. Ramadan studies have reported increased frequency of menstrual irregularities in lean women, particularly when fasting is prolonged, repeated over many days, or occurs under conditions of dehydration and environmental stress [155]. However, these observations are heterogeneous and often confounded by concurrent changes in sleep timing, hydration, diet composition, and physical activity, limiting causal interpretation.

Across these contexts, a consistent integrative principle emerges: the primary determinant of IF tolerability in reproductive-age women is overall energy availability relative to physiological demands—including menstrual cycling, physical activity, and psychosocial stressors—rather than the mere presence of fasting windows. This is supported by the concept of energy availability, defined as dietary energy intake minus exercise energy expenditure normalized to fat-free mass, which is a key regulator of reproductive function [156]. When energy availability falls below approximately 30 kcal/kg fat-free mass per day, disruptions in GnRH and LH pulsatility, reductions in estradiol, and menstrual disturbances may occur even in the absence of overt weight loss [157]. Mechanistically, low energy availability is characterized by reductions in leptin and insulin, elevations in cortisol, and impaired kisspeptin signaling, all of which converge at the level of hypothalamic GnRH neurons [158,159]. This framework provides a unifying explanation for how fasting may interact with exercise load, dietary restriction, and baseline metabolic status to influence reproductive outcomes.

Within this context, appetite-regulating hormones further contribute to the observed variability in responses to IF. Short-term fasting has been shown to reduce leptin concentrations, with more heterogeneous effects on adiponectin and ghrelin, changes that closely track negative energy balance [160]. Given leptin’s role as a permissive signal for GnRH secretion, fasting-induced reductions in leptin may contribute to the observed suppression of LH pulsatility, even when ovulation is preserved in the short term [154,161]. Combined interventions involving IF and exercise may further amplify these signals, potentially increasing the risk of low-energy-availability states in active women [161].

Menstrual cycle phase also influences behavioral adherence and perceived tolerability. Daily energy intake is typically lower in the follicular phase and higher in the luteal phase, with differences of approximately 150–500 kcal/day [162,163]. In addition, ghrelin concentrations and subjective hunger tend to be higher in the luteal phase, while anorexigenic GLP-1 responses may be blunted, creating a hormonal milieu less favorable for prolonged fasting [164]. These cyclical changes provide a physiological basis for the observation that women may tolerate longer fasting windows more easily during the early-to-mid-follicular phase, whereas the luteal phase may be associated with greater difficulty adhering to restrictive eating schedules. Although this hypothesis has not yet been directly tested in IF trials, it supports recent methodological recommendations to incorporate menstrual phase tracking when prescribing or studying fasting interventions in women [158,165], and suggests a more flexible, phase-aware approach in clinical practice [166].

Baseline metabolic phenotype and physical activity further modulate these responses. In women with overweight or obesity, insulin resistance, metabolic syndrome and polycystic ovary syndrome (PCOS), IF and TRE can induce modest weight loss, reduce waist circumference, improve fasting glucose and insulin sensitivity, and favorably alter triglycerides and other cardiometabolic markers, often with neutral or even beneficial effects on menstrual regularity over short-term interventions [167,168,169]. In contrast, similar fasting protocols applied to lean, insulin-sensitive women may result in proportionally greater reductions in energy availability, increasing the likelihood of fatigue, menstrual disturbance, or low-energy-availability symptoms [170,171,172]. The interaction with physical activity is particularly relevant in active and athletic women. While some studies combining IF with exercise report improvements in body composition and physical performance [173,174,175] sports science literature consistently remark the high prevalence of low energy availability and related symptoms in female athletes [176,177]. Compressing energy intake into restricted windows may therefore increase the risk of under-fueling around key training sessions if not carefully managed. Accordingly, adequate energy and macronutrient intake, particularly around exercise, should be prioritized when IF is implemented in physically active women.

Finally, fasting interacts with broader endocrine systems, including thyroid and stress axes. Prolonged energy restriction is associated with reduced T3 levels, increased reverse T3, and adaptive reductions in metabolic rate [158], and serum T4 and T3 concentrations may decrease in the last days of Ramadan in women [178]. Likewise, acute fasting stimulates activation of the hypothalamic–pituitary–adrenal (HPA) axis and cortisol secretion [17]. Some evidence suggests that women may exhibit greater HPA-axis responsiveness to metabolic stressors [179,180], and alterations in cortisol rhythms have been reported in different fasting contexts [181]. This chronic elevation or dysregulation of cortisol may further contribute to metabolic and reproductive disturbances, particularly in women exposed to high baseline stress or sleep disruption [182].

##### Preconception and Fertility Considerations

For women actively trying to conceive, preserving regular ovulation and adequate luteal function remains the primary consideration when contemplating IF. Some preclinical models have recently reported that IF can reverse the aging-associated decline in oocytes through various mechanisms, thus improving the fertility of aged female mice [183]. However, to date, there are no randomized controlled trials directly evaluating IF as fertility interventions in normo-ovulatory women or in those undergoing ovulation induction or in vitro fertilization (IVF), and available data are largely indirect, stemming from PCOS cohorts in whom TRE has improved hyperandrogenism, insulin resistance, and menstrual cyclicity—changes that may plausibly support improvements in reproductive function but do not yet confirm enhanced fecundity [167].

One prospective cohort study conducted during Ramadan provides limited but informative human data in the assisted reproduction context [184]. In this study, 300 fasting infertile women undergoing IVF/ICSI were compared with 300 matched non-fasting controls during the same period. Live birth rates did not differ significantly between groups, and embryo quality was comparable, suggesting that short-term religious fasting did not substantially impair IVF outcomes. However, women in the fasting group required higher doses and longer durations of ovarian stimulation, and pregnancy complications were more frequent among successful pregnancies. Interestingly, fasting women reported significantly lower levels of anxiety and depression during treatment. Although these findings suggest that Ramadan fasting does not markedly compromise IVF success rates, the observational nature of the study, the unique behavioral context of Ramadan (including changes in sleep, hydration, and meal timing), and the absence of controlled IF interventions limit causal interpretation. Consequently, these data should be interpreted cautiously when extrapolating them to structured IF regimens.

Preconception nutrition studies in IVF populations [185,186] emphasize overall dietary patterns rather than meal timing, with higher adherence to cardiometabolic-friendly dietary patterns (e.g., Mediterranean-style)associatedg with lower pregnancy loss but not consistently with higher live birth rates, indicating that quality and adequacy of intake may be more critical for fertility outcomes than specific fasting windows.

Given the well-established sensitivity of ovulatory function to low energy availability and psychological stress, aggressive intermittent fasting regimens (e.g., ADF, PDF, or fasting combined with substantial caloric restriction) should be approached with caution in women attempting pregnancy, particularly in those with infertility, prior functional hypothalamic amenorrhea, low body weight, high exercise loads, or other indicators of reproductive vulnerability. If IF is considered at all, a conservative approach—such as mild TRE with stable weight, adequate energy and micronutrient intake, and monitoring of cycle characteristics and ovulatory markers—appears more prudent than more restrictive protocols. However, this recommendation is based largely on indirect evidence and physiological reasoning rather than on direct fertility trials. Until prospective studies specifically examine ovulation frequency, luteal phase adequacy, Anti-Müllerian Hormone, antral follicle count, and conception or IVF outcomes under intermittent fasting regimens, any use of fasting in women attempting pregnancy should be individualized and framed as experimental rather than evidence-based care.

#### 3.1.2. Pregnancy and Lactation

Pregnancy profoundly alters maternal fuel partitioning, with differential changes depending on the phase. During early pregnancy, placental hormones promote increased food intake, enhanced insulin secretion, and anabolic energy storage, leading to expansion of maternal adipose reserves that support later fetal growth and lactation [187]. In contrast, late pregnancy is characterized by a shift toward insulin resistance and increased lipolysis, elevating maternal glucose and lipid availability to facilitate continuous nutrient transfer—particularly glucose—to the developing fetus [188].

Most available human evidence regarding fasting during pregnancy derives from Ramadan-style dawn-to-sunset fasting, rather than from structured IF regimens. Early metabolic observations in rural West African women showed that late-pregnancy fasting during Ramadan was associated with increased FFAs and β-hydroxybutyrate and reduced alanine concentrations, consistent with reduced maternal capacity to maintain euglycemia during prolonged fasting [189]. Ketone bodies readily cross the placenta and can serve as substrates for fetal brain development. Contemporary reviews therefore consider moderate maternal ketonemia a physiological adaptation, but sustained or excessive exposure has been associated in some older cohorts with lower offspring cognitive scores or abnormal fetal surveillance patterns, particularly in pregnancies complicated by diabetes or maternal undernutrition, although causality remains uncertain [190,191]. Consequently, clinical guidelines generally recommend minimizing recurrent maternal hypoglycemia and marked ketonemia during pregnancy, especially in women with pre-existing or gestational diabetes in whom prolonged fasting may precipitate both metabolic disturbances [192].

Despite this, a recent umbrella review concluded that Ramadan fasting during pregnancy is not consistently associated with major adverse effects on birthweight or most obstetric outcomes, although the certainty of evidence remains low due to substantial heterogeneity across studies [193]. Similarly, a large systematic review and meta-analysis including more than 30,000 pregnancies found no clear association between Ramadan fasting and preterm birth, hypertensive disorders of pregnancy, or major perinatal complications, although placental weight appeared slightly lower among fasting mothers and data on perinatal mortality were limited [194]. Despite this, focused fetal-outcome reviews and observational cohorts have described modest associations with lower neonatal birthweight, reduced amniotic fluid index, or altered fetal growth parameters—particularly when fasting occurs during the second or third trimester or when maternal nutritional intake during non-fasting hours is suboptimal [195,196]. A recent cohort additionally observed a greater birthweight reduction in the offspring of women fasting during Ramadan in their first trimester of pregnancy and consuming low daily fat, whereas for those in the second and third trimester the combination of fasting with a reduction in the intake of fluids and reduced sleep was associated with a lower birthweight [197]. Not all the studies have reported negative associations between Ramadan fasting and pregnancy outcomes. For instance, Safari et al. [198] reported that fasting during the second trimester of the pregnancy decreased the risk of gestational diabetes and excessive weight gain during pregnancy. However, these findings remain inconsistent and are largely derived from observational studies with limited control for different critical variables potentially affecting these results.

Importantly, Ramadan fasting differs substantially from deliberate non-religious IF regimens such as prolonged TRE or ADF, and the existing literature therefore cannot be directly extrapolated to IF protocols during pregnancy. Moreover, the absence of consistent adverse effects on birthweight should not be interpreted as proof of safety, as most studies are underpowered to detect rare but clinically significant outcomes such as miscarriage, stillbirth, congenital anomalies, maternal hypoglycemia, or long-term neurodevelopmental and cardiometabolic effects in the offspring [194,196]. Risk also seems likely to be modified by gestational age, fasting duration and season, climate, maternal nutritional status, workload, and the presence of diabetes or other obstetric complications [194,196]. Accordingly, pregnancy is generally considered a physiological state in which deliberate non-religious IF should be discouraged, and decisions regarding religious fasting should be individualized, based on careful risk stratification and appropriate medical counseling.

During lactation, evidence on fasting—primarily derived from Ramadan studies and limited observations of short-term religious fasting such as Yom Kippur—suggests that diurnal fasting does not substantially alter human milk macronutrient composition (fat, protein, lactose, or total energy) in healthy and well-nourished women, and short-term infant growth appears unaffected [199,200]. Recent analyses confirm similar milk energy, protein, carbohydrate, and lipid content between fasting and non-fasting lactating mothers, though some micronutrient profiles (e.g., electrolytes, minerals like zinc or magnesium) may show moderate changes, particularly with chronic repetitive fasting or suboptimal maternal intake during eating windows [199,200].

Although well-nourished women who undertake short periods of religious fasting may maintain adequate milk production, regional consensus statements and clinical reviews nonetheless caution that, in undernourished women, those with high physical workloads, or when fasting exceeds ~20–24 h or is combined with caloric restriction, maternal reserves and potentially milk quality or volume may be compromised, warranting monitoring [199,200]. For these reasons, deliberate IF for weight loss is generally not recommended during breastfeeding, particularly in the early postpartum period when maternal energy requirements increase [201,202]. In addition, experimental evidence from animal models suggests that maternal dietary restriction during lactation—whether through CR or IF—can lead to sustained undernutrition in offspring, accompanied by reductions in circulating glucose levels and alterations in hypothalamic redox balance, persisting into adolescence [203]. These findings highlight the critical role of adequate maternal energy intake during lactation and raise concerns that restrictive dietary patterns may adversely affect offspring metabolic development and neurobiological outcomes, particularly under conditions of insufficient maternal nutritional reserves. Lactating women who choose to fast for religious reasons should receive counseling regarding adequate fluid and nutrient intake during non-fasting hours and be monitored for signs of reduced milk supply or maternal depletion.

#### 3.1.3. Menopause and Postmenopausal Metabolic Adaptations

Menopause triggers profound metabolic shifts characterized by estrogen decline, which promotes visceral adipose tissue accumulation, insulin resistance, and heightened metabolic syndrome risk [204]. Estrogen deficiency also alters energy expenditure, mitochondrial function, and lipid metabolism, contributing to increased circulating FFAs and chronic low-grade inflammation—key factors underlying the elevated cardiometabolic risk observed in postmenopausal women [205]. Postmenopausal women exhibit reduced *de novo* lipogenesis in subcutaneous fat alongside compensatory vascular endothelial growth factor expression in visceral depots, exacerbating ectopic lipid deposition and insulin signaling impairment [206].

IF may partially counterbalance these changes by improving insulin sensitivity, enhancing lipid oxidation, and promoting mobilization of visceral fat stores, partly through reductions in fasting insulin exposure [207]. In addition, IF may attenuate menopause-associated oxidative stress and chronic inflammation and improve circadian metabolic regulation, suggesting a potential role in restoring metabolic homeostasis in estrogen-deficient states, even in the absence of substantial weight loss [208,209].

Human studies primarily randomized controlled trials of time-restricted eating TRE and ADF have reported reductions in body weight, fat mass, and insulin resistance comparable to those observed in premenopausal women. For example, an 8-week TRE trial (4–6 h eating window) yielded approximately 3.3% weight loss, accompanied by fat mass reduction, improved insulin sensitivity (lower HOMA-IR), and reduced oxidative stress (8-isoprostane), with similar responses in pre- and postmenopausal women [210]. Similarly, pooled analyses of ADF trials (~12 weeks) indicate weight reductions of approximately 5–6%, together with improvements in insulin resistance and blood pressure across both pre- and postmenopausal populations, with some studies reporting greater reductions in LDL cholesterol among postmenopausal participants [149].

Additional small trials in postmenopausal women with obesity or inflammatory conditions have also reported improvements in body composition and reductions in inflammatory markers following 16:8 TRE protocols. In one recent randomized trial, combining TRE with resistance training in postmenopausal women led to greater improvements in skeletal muscle mass, insulin sensitivity, and lipid profiles compared with resistance training alone [211]. Beyond metabolic outcomes, emerging evidence suggests that lifestyle interventions incorporating intermittent fasting may also influence cognitive health in this population. In a randomized controlled trial [212] involving postmenopausal women with obesity, a dietary intervention based on IF two days per week was compared with physical-cognitive exercise, a combined intervention, and a control group over three months. While IF alone improved body composition and cardiometabolic parameters—including reductions in cholesterol levels and improvements in anthropometric indices—no significant improvements in cognitive outcomes were observed in the diet-only group. By contrast, the combined intervention (fasting plus exercise) produced significant improvements in executive function, accompanied by increases in circulating brain-derived neurotrophic factor (BDNF) and adiponectin levels as well as reductions in insulin resistance and body fat [212]. These findings suggest that IF may contribute to cardiometabolic improvements in postmenopausal women, but that synergistic effects with structured exercise may be required to achieve measurable cognitive benefits.

Currently, there are more studies comparing TRE, TRE plus resistance training and CR plus resistance training in overweight and obese postmenopausal women in order to deepen and compare the precise benefits of each intervention [213]. Meta-analyses of IF interventions in overweight and middle-aged populations generally report modest body-weight reductions (~3–7%) together with improvements in several cardiometabolic risk markers, including fasting insulin, lipid profile, and blood pressure [214,215,216]. However, postmenopausal subgroup analyses remain limited, as most trials include mixed populations without menopause-specific stratification.

Despite these promising findings, the current evidence base remains constrained by small sample sizes, short intervention durations, and limited sex- or menopause-specific analyses. Most studies include fewer than 50 participants per group and follow participants for only 8–12 weeks, making it difficult to assess long-term sustainability, safety, or effects on menopause-specific outcomes such as visceral adiposity, sarcopenia, or mitochondrial function. Moreover, few trials are specifically designed for postmenopausal women. Larger and longer-duration randomized studies focusing on this population are therefore needed to clarify the long-term metabolic and cardiovascular implications of IF in the postmenopausal period.

**Figure 5:**
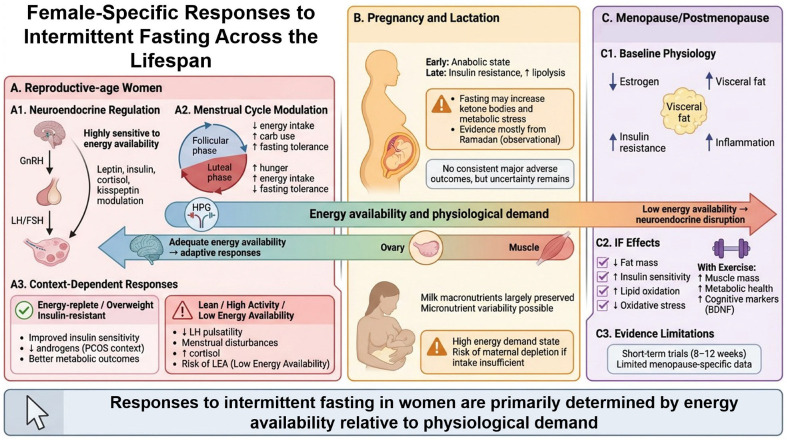
Female-specific considerations of intermittent fasting across the lifespan. The effects of intermittent fasting (IF) in women are largely determined by energy availability relative to physiological demand and vary across life stages. In reproductive-age women, responses are highly dependent on neuroendocrine regulation and menstrual cycle phase, with increased risk of low energy availability and menstrual disturbances in lean or highly active individuals. During pregnancy and lactation, elevated physiological demands and limited evidence warrant caution, despite generally neutral findings from Ramadan-type fasting. In menopause, IF may improve metabolic health, although evidence remains limited. Overall, tolerance and outcomes of IF in women are context-dependent, emphasizing the importance of individualized approaches. Abbreviations: IF, intermittent fasting; LEA, low energy availability. ↑: increase; ↓: reduction.

In Figure 5, we summarize the main considerations regarding the female-specific responses to IF across the lifespan.

### 3.2. Specific Considerations of Intermittent Fasting in Men

Men may show some distinct responses to IF partly because of differences in baseline body composition, androgen status, and adipose tissue distribution. As previously stated, men generally have greater lean mass and a higher tendency toward visceral fat accumulation compared to women [217], factors that may influence both metabolic and endocrine responses to fasting. Current evidence suggests that these sex-related characteristics may shape the effects of IF on body composition and hormonal regulation, although the number of male-specific studies remains limited [214,218].

One of the most discussed male-specific issues is the potential effect of IF on testosterone. A 2022 review of randomized trials concluded that specific IF regimens such as TRE consistently reduced total and free testosterone concentrations in lean, physically active, young men, while SHBG concentrations generally remained unchanged; importantly, these hormonal reductions were not accompanied by clear impairments in muscle mass or strength in the short to medium term [148]. Similar findings have been reported in individual resistance-training studies, including 12-month follow-up data, in which testosterone declined despite preservation of muscle performance and improvements in several metabolic and inflammatory markers [219]. A classical study [220] reported that in healthy young men, a five-day fast significantly reduces testosterone and LH levels by decreasing the amount of LH released per secretory burst, suggesting that nutrient deprivation impairs testosterone production by weakening hypothalamic GnRH signals rather than changing the frequency of hormone release. Conversely, in men with obesity, 12 months of TRE promoted weight loss without affecting testosterone, dehydroepiandrosterone (DHEA), and SHBG [221]. These observations suggest that fasting may modestly lower circulating testosterone in some men, particularly in lean and active populations, although the clinical significance of this effect remains uncertain [222].

Additional endocrine adaptations may also contribute to men’s physiological responses to IF. As in other forms of energy restriction, IF can influence the HPA and thyroid axes through changes in energy availability and metabolic signaling. Acute fasting, particularly early TRE and long periods of fasting appears to increase cortisol secretion in men according to previous studies, reflecting the mobilization of endogenous energy substrates and the activation of stress-responsive metabolic pathways [158,220,223,224]. While these responses are generally transient and considered physiologically adaptive, prolonged or aggressive fasting combined with high training loads may theoretically contribute to fatigue, sleep disruption, or impaired recovery in physically active men [225].

Thyroid function may also adapt to fasting-induced energy restriction. As for women, during prolonged fasting, circulating T3 and free T3 (FT3) levels typically decline, while the inactive metabolite rT3 increases [158]. However, no significant alterations in serum concentrations of T4, T3, TSH, and TSH response to of TRH stimulation were found in fasting Muslim males [178]. These changes are generally interpreted as adaptive metabolic adjustments rather than pathological thyroid dysfunction. In men undertaking IF for metabolic or body-composition purposes, these endocrine adaptations likely reflect the organism’s attempt to optimize energy utilization during periods of reduced caloric intake.

From a body-composition perspective, IF combined with exercise appears to be reasonably well tolerated in men and may help reduce fat mass while preserving lean tissue. Systematic reviews of IF plus resistance training report that lean body mass is generally maintained, whereas fat mass often declines [82]. Individual trials in resistance-trained men have likewise shown reductions in fat mass and waist-related measures without clear decrements in strength or muscle performance [226]. Accordingly, the main practical concern in men may not be loss of muscle per se but rather ensuring sufficient energy and protein intake to avoid unnecessary reductions in anabolic signaling during prolonged or more aggressive fasting protocols.

In men with overweight or obesity, IF is more consistently associated with modest reductions in body weight, waist circumference, and several cardiometabolic risk markers, including fasting insulin, blood pressure, and lipids, although most evidence derives from mixed-sex trials rather than male-specific cohorts [214,227].

Finally, the implementation of IF to improve male fertility remains preliminary and exploratory, with no large-scale randomized controlled trials confirming efficacy as of 2026. A 2025 mixed-methods RCT (n = 18 completers) tested three 5-day FMDs cycles (500 kcal/d) versus waiting-list control in subfertile men with impaired sperm quality [228]. While no statistically significant group differences emerged, positive trends favored FMD for total/progressive motility, concentration, morphology, and reduced round cells. Contrasting data from Ramadan fasting studies show transient declines in progressive motility and semen volume, suggesting potential short-term risks [229]. Preclinical models indicate that IF may restore high-fat diet-induced fertility deficits via SIRT1/NRF2 upregulation [230], but human translation requires robust, powered trials to assess sperm parameters, hormones, and live birth outcomes before clinical endorsement.

**Figure 6:**
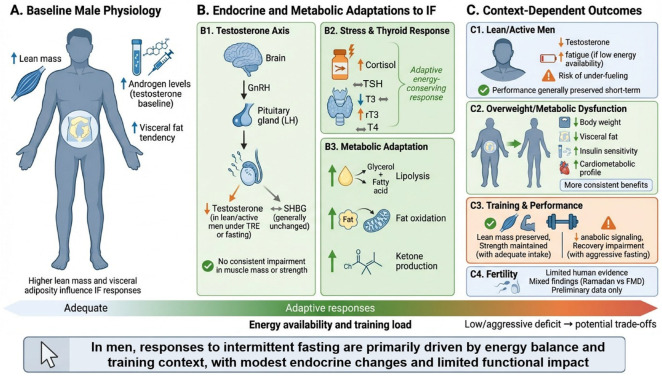
Male-specific metabolic, endocrine, and performance-related responses to intermittent fasting. In men, responses to intermittent fasting (IF) are influenced by baseline physiology, including higher lean mass, greater visceral adiposity, and androgen status. IF induces adaptive endocrine and metabolic changes, including modest reductions in testosterone in lean and physically active men, typically without consistent impairments in muscle mass or strength, alongside increases in cortisol (acute/adaptive) and possible shifts or no variations in thyroid hormones consistent with energy conservation (↓ T3, ↑ rT3). These changes are accompanied by enhanced lipolysis, fat oxidation, and ketone production. Outcomes are strongly context-dependent. In lean or highly active men, aggressive fasting or low energy availability may increase fatigue and under-fueling risk, although short-term performance is generally preserved. In contrast, men with overweight or metabolic dysfunction tend to experience more consistent improvements in body composition and cardiometabolic health. When combined with resistance training and adequate energy and protein intake, IF can support fat loss while preserving lean mass and strength, whereas more restrictive approaches may impair anabolic signaling and recovery. Evidence regarding male fertility remains limited and inconclusive. Overall, this framework highlights that responses to IF in men are primarily determined by energy balance and training context, with modest endocrine changes that rarely translate into functional impairment. Abbreviations: IF, intermittent fasting; TRE, time-restricted eating; rT3, reverse triiodothyronine. ↑: increase; ↓: reduction.

Thus, in men, IF may currently be viewed less as a targeted endocrine intervention and more as a potentially useful cardiometabolic strategy whose hormonal effects—particularly on testosterone—should be interpreted in relation to baseline leanness, training status, age, and overall energy balance. In Figure 6, we summarize the main considerations of IF.

To further contextualize these sex-specific differences and their clinical implications, Table 1 summarizes the main potential benefits, reported risks, and population groups in which intermittent fasting may be more appropriate or should be approached with caution. This integrative overview complements the mechanistic framework presented above and highlights the importance of tailoring fasting strategies according to biological sex, life stage, and energy availability.

## 4. Sex Differences in Physiological, Clinical and Psychological Responses to Intermittent Fasting

### 4.1. Exercise Performance

IF can generally be combined with exercise without abolishing training adaptations in either men or women, provided that total energy and protein intake are sufficient and that feeding is appropriately aligned with training demands [231,232]. However, direct sex-comparative evidence remains extremely limited. Most available studies were not designed or powered to detect sex-by-diet interactions, and current interpretations therefore rely largely on indirect comparisons across male-only, female-only, and small mixed-sex cohorts. This limitation is particularly relevant because subtle sex-specific differences in endocrine regulation, perceived energy, recovery, and vulnerability to LEA may remain undetected when sex is treated merely as a covariate rather than a primary analytical variable.

The strongest evidence comes from TRE usually in 16:8 or similar formats, combined with resistance training in recreationally active or trained individuals. Under these controlled conditions, both men and women appear capable of preserving or improving strength and lean mass while reducing fat mass [82]. In resistance-trained women, short-term TRE interventions with matched protein intake and structured training have generally shown maintained gains in fat-free mass, muscle thickness, and strength, together with modest reductions in fat mass and no major deterioration in resting metabolic rate or standard metabolic markers [175]. Parallel studies in men report a broadly similar performance and body-composition profile, with maintained lean mass and strength despite reductions in fat mass [233]. Taken together, these data suggest that, in the short to medium term, TRE does not inherently impair resistance training adaptations in both sex when dietary intake is carefully controlled.

Notably, recent meta-analytic evidence [234] incorporating subgroup analyses by sex suggests that combining IF or CR with exercise does not significantly affect VO_2_max in either women or men, while inducing comparable reductions in body weight and fat mass in both sexes. However, sex-specific patterns may emerge for lean mass, with significant reductions in fat-free mass observed in men but not consistently in women [234]. Despite these subgroup findings, the included studies were not primarily designed or powered to detect sex-by-intervention interactions, sample sizes were uneven, and the number of female-specific analyses remained limited. Therefore, these results should be interpreted cautiously and do not yet establish true sex equivalence in physiological responses to IF combined with exercise.

Where men and women may diverge is less in overt performance outcomes than in their hormonal and physiological responses to the same fasting stimulus. In men, TRE combined with resistance training has repeatedly been associated with reductions in total testosterone and, in some studies, IGF-1, despite preserved muscle mass and strength [226]. By contrast, equivalent resistance training studies in women have not shown a comparably consistent signal of impaired anabolic adaptation [235], although menstrual-related outcomes and cycle phase are seldom rigorously characterized. Thus, the current literature suggests that both sexes retain the capacity to adapt to resistance training under IF protocols like TRE, but that the endocrine correlates of these adaptations may differ, with hormonal downregulation more consistently documented in men and female-specific reproductive endpoints remaining underexplored rather than clearly unaffected.

A very recent, small mixed-sex trial in well-trained adults conducted by Blake et al. [233] is particularly informative because it approximates the type of design needed for meaningful comparison. In that study, both men and women completed progressive resistance training under either 16:8 TRE or a conventional feeding schedule with high protein intake and a mild caloric surplus. Both groups improved strength and fat-free mass, and TRE attenuated fat-mass gain; however, participants assigned to TRE achieved slightly lower training volume and somewhat smaller improvements in squat one-repetition maximum. Importantly, the study did not identify clear sex-specific effects, but the sample size was very small, and sex was not treated as the main variable of interest. Accordingly, the absence of a detected sex difference should not be interpreted as evidence of true equivalence.

Evidence for endurance exercise is substantially weaker and remains heavily male-dominated. In trained men, short-term 16:8 TRE does not appear to impair VO_2_max, running economy, or time-trial performance, while modestly increasing fat utilization during submaximal exercise [236]. Supporting this, an 8-week study in recreationally active adults—mostly women—showed that FATmax training improved fat oxidation independently of whether exercise was performed in the fasted or fed state, suggesting that no additional or synergic benefits from IF on fat oxidation can be observed under well-designed [237]. Similarly, Stannard et al. [238] observed in earlier mixed-sex data that endurance training adaptations occur in both fasted and fed conditions, with greater VO_2_max improvements under fasted training but no clear sex × intervention interaction. However, in a subsequent exploratory analysis, they suggested that men may respond slightly better to fasted training, whereas women may adapt more favorably in the fed state [238]. Overall, the lack of adequately powered female-specific or sex-comparative studies limits firm conclusions, and it remains unclear whether women exhibit the same tolerance or metabolic flexibility as men under IF combined with endurance training.

Ramadan fasting offers an additional, more ecologically valid model of intermittent fasting in athletic populations. Across existing studies, performance decrements are usually small and most evident in repeated-sprint or very high-intensity tasks, particularly when sleep disruption and circadian misalignment coexist [238]. Meta-analytic evidence [239] indicates that Ramadan fasting may induce small, transient reductions in aerobic capacity (e.g., VO_2_max), especially during the early phases, likely related to dehydration, reduced glycogen availability, and altered sleep patterns; however, these effects tend to attenuate as the fasting period progresses. Both male and female athletes often maintain overall aerobic and neuromuscular performance reasonably well across the fasting period [240], although transient reductions in body mass and energy intake are common. The available data do not demonstrate systematically greater performance losses in women than in men during Ramadan-type fasting [241], but female cohorts remain small and study designs are often confounded by changes in sleep, hydration, and training timing. Therefore, Ramadan findings should be interpreted as supportive but not definitive evidence regarding sex differences.

With respect to substrate utilization, IF combined with exercise appears to favor greater reliance on fat as a fuel source in both sexes, especially during submaximal exercise [242,243]. This shift is more consistently documented in male endurance studies [244], whereas in women the available data suggest either modest changes or preservation of baseline substrate use under controlled TRE plus resistance training [175]. This difference may reflect well-established sex-specific metabolic patterns, whereby men tend to rely more on carbohydrates to sustain moderate aerobic exercise, whereas women exhibit a greater reliance on lipid metabolism [245]. Indeed, fasting studies indicate that women display higher lipolytic rates, greater circulating FFAs, and a more pronounced reduction in plasma glucose compared to men, particularly as fasting duration increases, reflecting a stronger shift toward lipid utilization [14]. *In vivo* models further support this concept, suggesting that males and females may achieve similar phenotypic outcomes when combining IF plus high-intensity interval training (HIIT) through distinct molecular pathways, with males favoring enhanced fat oxidation and females reduced lipid storage [246]. These mechanistic differences may contribute to the observed variability in substrate utilization responses under IF conditions. Thus, although the direction of adaptation seems broadly similar, the magnitude and practical relevance of these metabolic shifts remain uncertain in women because of the paucity of dedicated studies.

Differences in subjective energy, fatigue, and recovery may be especially important in real-world implementation. Even when objective performance is preserved, some mixed-sex IF studies report lower perceived daily energy and reduced achieved training volume under TRE [233]. These effects do not appear large enough to abolish adaptation over short study periods, but they may become more relevant over longer training blocks or in athletes with high workloads. In women, this issue warrants particular caution because compressed eating windows may make it more difficult to distribute carbohydrate and protein intake optimally around training, especially under conditions of high expenditure or pre-existing vulnerability to LEA. Since these broader risks have already been discussed in the previous sections, the key point here is that IF may be physiologically compatible with training in both sexes under controlled conditions, yet the practical burden of achieving adequate fueling may be disproportionately greater in women in free-living athletic settings.

Importantly, the strength of evidence underlying these observations varies across outcomes. While resistance training adaptations are supported by controlled human studies, evidence for endurance performance and other domains remains more limited and often derives from small mixed-sex cohorts, secondary analyses, or predominantly male-based studies. Accordingly, sex-specific interpretations in these areas should be considered provisional and interpreted with caution.

### 4.2. Management of Chronic Diseases

IF has been examined as an adjunctive strategy in several chronic disease settings, but sex-specific clinical evidence is sparse, often indirect, and heavily weighted toward women in some domains (e.g., postmenopausal rheumatoid arthritis, breast and gynecologic cancers) while underrepresenting women in others (e.g., cardiometabolic trials in mixed samples). In this section, we will summarize the current preclinical and human evidence regarding differential sex-dependent effects of IF in four groups of the most prevalent chronic maladies.

(A) Metabolic disorders

The global prevalence of obesity is rising at an alarming rate, with projections indicating that over 1.9 billion adults will be affected by 2035, necessitating highly effective and personalized lifestyle interventions [114]. Significant inter-sexual differences define the landscape of metabolic syndrome; men typically exhibit higher BMI and fat-free mass index (FFMI), alongside a predisposition for visceral adiposity, which is more strongly associated with systemic inflammation and insulin resistance [247]. In contrast, premenopausal women often present with higher fat mass index (FMI) but tend to store lipids in subcutaneous gluteofemoral depots, which may offer a degree of metabolic protection [248]. At the baseline fasting level, females demonstrate lower mean concentrations of plasma glucose and lactate and a lower resting metabolic rate (RMR), while conversely exhibiting higher concentrations of plasma insulin and free fatty acids compared to males [144]. These baseline variations suggest that the “metabolic switch” from glucose to fatty acid oxidation during fasting may be initiated from fundamentally different physiological starting points.

Preclinical work in obese and metabolically challenged rodents consistently shows that females often derive equal or greater metabolic benefit from IF or TRE than males, especially in terms of insulin sensitivity, energy expenditure, and inflammatory markers [249,250]. In more detail, rodent models that directly compare obese males and females exposed to the same IF or intermittent energy restriction protocol, report that females tend to show larger reductions in HOMA-IR, more robust upregulation of hepatic insulin-signaling pathways (e.g., IRS2, Sirt1), and greater decreases in pro-inflammatory cytokines in subcutaneous and visceral adipose depots than males, even when weight loss is similar [250,251,252]. Some of these models also demonstrate that only females increase sympathetic innervation of brown adipose tissue and maintain higher total energy expenditure under IF, whereas males exhibit a relative decline in energy expenditure and persistently higher leptin levels for a given degree of weight loss [249]. Conversely, Chaix et al. [253] reported in C57BL/6J mice consuming a Western diet that 9 h TRE improved fatty liver and glucose intolerance in both sexes, but body-weight benefit was observed primarily in males. These results suggest sex-dependent mechanisms (BAT activation, leptin–adipose signaling, adipose inflammation) that could translate into different cardiometabolic trajectories in men versus women if replicated in humans.

However, translation of these advantages into human sex-differentiated clinical outcomes remains limited by small samples, lack of prespecified sex-intervention analyses, and short follow-up. Meta-analyses and umbrella reviews [6] indicate IF produces modest weight loss and improvements in some glycemic and blood-pressure metrics but generally does not outperform isocaloric CER for weight loss and many cardiometabolic markers, implying that the energy deficit and weight loss—rather than fasting per se—explains a substantial fraction of benefit in typical clinical implementations.

Within the ADF literature specifically, a pooled secondary analysis examining sex and menopausal status in adults with obesity reported that body weight decreased similarly in premenopausal women, postmenopausal women, and men, and that most metabolic risk markers improved comparably; a notable exception was a greater LDL-cholesterol reduction in postmenopausal versus premenopausal women, suggesting that hormonal life stage could modulate specific lipid endpoints even when weight loss is similar [149]. A major ADF randomized trial comparing ADF, daily calorie restriction, and control in adults with obesity found no superiority of ADF for weight loss and documented substantial dropout—highlighting adherence and tolerability as major determinants of real-world effectiveness, which could plausibly vary by sex and life stage but are seldom analyzed in that way [72]. For TRE, controlled trials demonstrate mild weight loss and improvements in insulin resistance and oxidative stress over short periods (e.g., ~8 weeks) under narrow eating windows; these trials have typically reported pooled effects rather than robust sex-by-intervention analyses, even when both sexes are enrolled [254].

A 6-month randomized trial in adults with type 2 diabetes found that TRE produced greater weight loss than continuous CR, with similar improvements in HbA1c across diet arms, but the results were not reported separately for men and women, and the trial was not powered to detect sex differences [255]. Another randomized trial in type 2 diabetes compared TRE to a standard dietary approach and included about 40% women; continuous glucose monitoring demonstrated improved mean glucose, time-in-range, and glycemic indices over six months, again without evidence of differential efficacy by sex because such analyses were not undertaken [256]. Ongoing trials such as the TEA TIME and related protocols explicitly plan sex-stratified analyses by capping female-to-male recruitment ratios, recognizing known sex differences in fat distribution and insulin sensitivity, but their outcome data are not yet available [257]. In humans, recent meta-analyses of TRE and other IF strategies confirm overall benefits on weight and cardiometabolic risk factors and show that percentage of female participants contributes to between-study heterogeneity in some risk markers in meta-regression [216,258], but the original trials rarely report sex-stratified outcomes, so these sex-related signals remain indirect and underexplored.

The principal gaps for sex-specific metabolic conclusions are therefore not only biological but methodological: (i) underpowered sex-stratified analyses; (ii) heterogeneous IF definitions (window length, early vs. late windows, fasting-day calories, macronutrient composition); (iii) limited follow-up for clinically hard endpoints (diabetes incidence, NAFLD histologic resolution, cardiovascular events); and (iv) insufficient integration of female reproductive life stage (menstrual cycle phase, perimenopause, menopause, hormonal contraception) into analysis plans, despite suggestive signals that menopausal status may modify specific lipid responses to ADF.

(B) Cardiovascular disease

Cardiovascular and vascular outcomes under IF have been less frequently analyzed by sex, despite clear sex differences in endothelial function and hormone–vascular interactions [259]. Observational data in healthy adults show that women generally have higher baseline flow-mediated dilation than men, with sex- and age-specific trajectories that are modulated by endogenous estrogen and menopause status, which are relevant for interpreting IF-induced vascular changes [260,261]. Preclinical evidence indicates that fasting regimens can produce either favorable or unfavorable vascular phenotypes depending on model, genotype, and dietary context, with sex sometimes explicitly tested. In Apoe−/− mice fed a Western diet, ADF increased atherosclerotic burden and worsened circulating cholesterol profiles [262]. To verify a possible sex-dependent effect, additional experiments were conducted in female Apoe−/− mice, showing similar pro-atherogenic effects. In this way, the authors argued that this adverse phenotype can occur in both sexes in this high-risk genotype/diet context. Conversely, other atherosclerosis-prone models report benefits from IF regimens in Apoe-deficient mice, improving dyslipidemia and atherogenesis, but they also found that these effects were irrespective of sex [263].

In humans, the cardiology outcome literature remains early-phase and focused largely on intermediate markers rather than clinical endpoints, limiting sex-differential inference. Currently, IF has been proven as an effective tool to promote weight loss and reduce multiple cardiovascular risk markers [264,265], whereas in other studies, neutral or even adverse effects have been reported [264]. In addition, the evidence does not clearly separate outcomes by sex, despite involving both men and women.

The major gaps are therefore (i) scarcity of adequately powered trials with prespecified sex-by-intervention interaction testing for vascular endpoints; (ii) limited evidence in female-specific high-risk windows (pregnancy-associated hypertensive disorders history, perimenopause/early postmenopause) where CVD risk evolves rapidly; (iii) uncertainty about whether lean-mass effects of IF and/or circadian misalignment (late eating windows) differ by sex and contribute to divergent long-term cardiovascular risk; and (iv) incomplete bridging between preclinical sex-inclusive atherosclerosis studies and human vascular endpoint trials. Methodological guidance emphasizing rigorous consideration of sex as a biological variable in vascular biology illustrates that the field recognizes these design challenges [266], but their application to IF studies remains uneven.

(C) Autoimmune and immune-inflammatory disorders

Sex differences in immune regulation are particularly relevant for autoimmune and inflammatory diseases. Females, on average, mount stronger humoral immune responses and have higher prevalence for many autoimmune diseases; mechanistic reviews implicate estrogenic effects on B-cell activation and autoreactivity, androgenic counter-regulation, and X-chromosome dosage/escape from inactivation among other factors [267]. These differences suggest that IF could, in principle, have sex-differential effects on autoimmune disease activity, both through endocrine-immune coupling and through immunometabolic remodeling.

In preclinical models, the benefits from IF in ameliorating autoimmune and inflammatory disorders have been broadly supported by the available evidence [268,269,270]. While these studies support biological plausibility for disease modification, many do not explicitly compare male versus female disease phenotypes under identical fasting protocols, or they use models with strong baseline sex bias in disease susceptibility, limiting clean attribution of “sex-specific fasting effects” versus “sex-different baseline disease.

Human evidence is suggestive but heterogeneous and often not sex-analytic. IF has been explored primarily in rheumatoid arthritis (RA) and multiple sclerosis (MS), with the most robust clinical data currently available in women with RA [271,272]. These data suggest that in estrogen-deficient, chronically inflamed women, IF may confer anti-inflammatory and antioxidant benefits without evident safety concerns over the short term; however, the absence of equivalent male cohorts under matched protocols precludes direct sex comparisons. In MS, early human work suggests feasibility and potential benefit of fasting/FMDs or ketogenic approaches in relapsing-remitting MS [273,274], but sex-specific evidence remains limited despite the clear female predominance of MS and known sex differences in immune biology [275].

The dominant limitations/gaps are: (i) underpowered sex-stratified analyses in human autoimmune IF trials (often small, single-center, short duration); (ii) intervention heterogeneity (water-only fasting vs. modified fasting vs. TRE/TRF vs. FMD, varying concomitant diets); (iii) disease heterogeneity (RA vs. MS vs. psoriasis vs. IBD) with distinct immunopathology and sex biases; and (iv) sparse translational immunophenotyping that is explicitly sex-stratified.

(D) Cancer

Sex differences in cancer are pervasive across incidence, survival, and therapy response, with males generally experiencing higher incidence and worse survival for many non-sex-specific cancers, and both hormone-dependent and hormone-independent mechanisms implicated (including sex chromosome effects, epigenetic programming, metabolism, and immunity) [276].

Preclinical data broadly support the concept that cyclic fasting/FMD can sensitize tumors to chemotherapy, targeted therapy, endocrine therapy, or immunotherapy while protecting normal tissues, often via reductions in circulating glucose/insulin/IGF-1 signaling and remodeling of immune infiltrates; however, many tumor-model studies remain single-sex or do not formally test sex-by-fasting interactions [277]. In humans, the strongest interventional signal comes from early-phase trials focused on feasibility, metabolic modulation, and toxicity rather than definitive survival endpoints. The DIRECT randomized trial [278] in HER2-negative early breast cancer tested an FMD around neoadjuvant chemotherapy and suggested improved radiologic/pathologic response in adherent patients, but compliance challenges and the single-sex disease context (breast cancer) limit generalization to sex-differential questions. A key mixed-tumor clinical trial of cyclic 5-day FMD in 101 cancer patients receiving standard therapies reported that FMD was safe/feasible, reduced glucose and growth-factor concentrations, and induced immunologic remodeling consistent with enhanced antitumor programs [279]. Notably, the enrolled cohort was predominantly female (73/101; 72.3%), with 28/101 (27.7%) male, and included a broad tumor spectrum (including prostate cancer as a male-specific disease within the cohort). Additional human studies—e.g., modified short-term fasting around chemotherapy—support feasibility and point toward metabolic and symptom/toxicity effects, but remain small and not designed to quantify sex-specific efficacy [277,280,281].

Thus, the cancer-specific “sex-differential IF” evidence base is constrained by: (i) tumor-type confounding (many IF trials focus on female-specific cancers, while mixed-cancer trials skew female); (ii) limited male-only IF oncology evidence outside of early cohorts in prostate cancer examining metabolic health endpoints; (iii) insufficient power and lack of prespecified sex interaction tests even in mixed cohorts; and (iv) the complexity of separating sex from gendered exposures, comorbidity patterns, and treatment differences in oncology outcomes. These limitations are increasingly recognized in oncology methodology and review literature emphasizing sex as a biological variable, but they remain a major barrier to concluding that IF has meaningfully different anticancer efficacy in women versus men [282].

### 4.3. Aging and Longevity Pathways

Aging and longevity are profoundly sexually dimorphic processes, and this biological background is essential for interpreting the potential effects of IF. Women outlive men across virtually all countries and historical periods, despite experiencing a higher burden of frailty and disability in later life, a phenomenon often referred to as the mortality–morbidity paradox [283,284,285]. This female survival advantage has been linked to multiple sex-specific mechanisms, including differences in sex chromosome biology, mitochondrial inheritance, hormonal regulation of immunity and inflammation, telomere dynamics, and distinct trajectories across several hallmarks of aging [286,287,288]. In line with this, molecular and cellular studies suggest that females often retain greater mitochondrial resilience and stress resistance, supporting the concept that aging biology itself is, among other factors, sex-dependent [289,290].

This sexual dimorphism seems to be reflected in responses to dietary restriction paradigms as well, including CR, TRE and other IF regimens. Preclinical evidence consistently indicates that lifespan and healthspan responses to dietary restriction vary by sex, although the direction and magnitude of the effect depend on the specific intervention, species, strain, age, and metabolic context [291]. In general, female rodents often show equal or greater metabolic benefit than males, particularly regarding insulin sensitivity, preservation of energy expenditure, adipose tissue remodeling, and inflammatory control. For example, in obese middle-aged mice, IF combined with caloric restriction improved weight-loss maintenance, preserved fat-free mass, increased energy expenditure, and reduced insulin and adipose inflammation more effectively in females than in males [249]. Similarly, female rodents under intermittent or continuous restriction have shown greater white adipose tissue loss and more pronounced improvements in insulin signaling than males [250]. These findings are consistent with broader observations that interventions targeting nutrient-sensing pathways, such as the GH/IGF-1 often extend lifespan more robustly in females [292,293].

At the same time, recent animal studies indicate that the effects of IF on longevity are not uniformly female-favoring, but rather highly context-dependent [294]. Large-scale preclinical work suggests that lifespan responses to IF are strongly influenced by genetic background, with some strains benefiting, others showing neutral effects, and some even being harmed by the same fasting protocol [107]. Moreover, IF may produce sexually dimorphic benefits through distinct mechanisms rather than identical pathways. For instance, recent experimental models suggest that males may respond preferentially through enhanced fat oxidation or altered myofiber remodeling, whereas females may respond through changes in lipid storage, oxidative muscle phenotype, or estrogen-sensitive hepatic pathways [295,296]. Emerging data also identify estrogen receptor α and hepatic ketogenesis as potential mediators of sex-specific TRE/IF responses, particularly in relation to hepatic lipid handling and fibrotic vulnerability [251,297].

Human evidence remains far more limited and is largely restricted to short-term metabolic outcomes rather than direct aging or longevity endpoints. To date, no clinical trial has examined sex-stratified effects of IF on validated biomarkers of biological aging, such as epigenetic clocks, telomere attrition, senescence markers, or long-term survival. Instead, available studies mostly evaluate body composition, glycemic control, lipid metabolism, and reproductive hormones over periods of weeks to months. Within this limited evidence base, some sex-related differences have emerged. Reviews of clinical IF studies suggest that men and women may differ in body-composition and glucose/lipid responses, possibly due to differences in sex hormones, fat distribution, and baseline metabolic phenotype [14]. Small human studies have reported greater reductions in BMI and fat mass in men than in women after short-term 16:8 IF [298] whereas other studies have shown broadly similar weight loss across males, premenopausal females, and postmenopausal females [149]. The comparison between the effects of IF in premenopausal and postmenopausal women have shown some mixed results, with some evidence supporting additional benefits of IF in postmenopausal women and others not reporting significant differences [210,235,299].

Overall, current evidence suggests that IF may influence aging-related pathways and metabolic health in both sexes, but its effects on longevity and healthspan are unlikely to be sex-neutral. Preclinical data generally support more favorable or at least distinct responses in females, especially in relation to nutrient-sensing pathways, adipose tissue remodeling, and inflammatory control, although these effects are heavily modified by genetic background, age, and the exact fasting regimen. In humans, however, evidence remains too limited to conclude whether IF confers differential benefits for aging or longevity in men versus women. Future research should therefore move beyond short-term metabolic endpoints and incorporate sex-stratified, age-aware, and menopause-sensitive designs, ideally including multi-omic profiling and validated biomarkers of biological aging, to determine whether fasting interventions can meaningfully and differentially modulate aging trajectories in women and men.

### 4.4. Psychological and Behavioral Responses

The psychological effects of IF are increasingly studied, yet the available evidence remains heterogeneous in quality, duration, and sex-specific reporting. Across the most common IF modalities, controlled trials in adult weight-management settings generally suggest neutral to small effects on mood, anxiety, perceived stress, sleep, and quality of life [300]. In structured interventions, IF does not appear to produce clinically meaningful deterioration in these psychosocial domains when implemented under supervision and accompanied by appropriate dietary guidance [88,300]. However, these reassuring findings coexist with a second body of evidence, largely observational, indicating that self-directed fasting practices are associated with greater eating-disorder (ED) psychopathology and disordered eating behaviors, particularly in adolescents, young adults, and individuals with pre-existing vulnerability [83]. This distinction between supervised clinical interventions and self-initiated fasting for weight control is critical when interpreting the psychological safety of IF.

Sex differences in psychological responses to IF are biologically plausible and likely clinically relevant. Appetite regulation, stress responsivity, reward processing, and sleep are all shaped by gonadal steroids, sex-specific neuroendocrine set points, and reproductive transitions such as the menstrual cycle and menopause [301,302,303,304]. Despite this, most IF trials treat sex as a descriptive covariate rather than a mechanistic variable, and very few account for menstrual-cycle phase, hormonal contraception, or menopausal status when assessing hunger, cravings, mood, or adherence, as claimed by the recent literature [305,306]. This omission is important because reproductive state may substantially influence subjective responses to fasting, particularly in women of reproductive age. Preclinical work supports this possibility, as animal studies have reported sex-dependent neurobehavioral responses to fasting paradigms, including differences in anxiety-like behavior, inflammation-linked depressive-like behavior, astrocytic remodeling, and neuroimmune signaling [307,308]. Together, these findings suggest that fasting can interact with affective and motivational systems in a sex-contingent manner, even though translation to humans remains incomplete.

In humans, the best-characterized psychological outcomes of IF concern subjective appetite and tolerability. Some randomized trials that collected repeated ratings of hunger, cravings, mood, and compliance have shown that hunger and cravings are predictably higher on fasting days than on non-fasting days, but that this does not necessarily translate into worsening mood [309]. Interestingly, the limited sex-disaggregated evidence suggests that men may report greater hunger overall than women in some IF protocols, whereas women may report lower hunger and cravings but also somewhat lower self-rated adherence [309]. This pattern is notable because it implies that subjective tolerability may not map neatly onto compliance, and that sex differences in appetite experience do not necessarily predict sex differences in psychological benefit or harm. More broadly, controlled TRE trials in adults with overweight or obesity have generally found no clinically relevant worsening in depression, anxiety, perceived stress, sleep quality, or quality of life in either men or women over intervention periods ranging from several weeks to 12 months [300,310]. Thus, in metabolically oriented RCTs enrolling generally healthy adults, IF appears psychologically neutral rather than clearly beneficial or harmful.

Sleep and cognitive outcomes are less consistent and remain under-characterized. Some studies suggest that IF may alter specific sleep dimensions in opposite directions—for example, improving subjective sleep quality or daytime function while worsening sleep latency or duration—indicating that “sleep” should not be treated as a unitary outcome [27,311,312]. Similarly, experimental studies of acute fasting suggest that attentional control may be transiently impaired when hunger becomes salient, although broader executive functioning does not appear uniformly disrupted [312]. Overall, controlled human data do not support robust cognitive enhancement with IF in healthy populations, despite some mechanistic evidence suggesting potential benefits in this sense, particularly when combined with other strategies such as physical activity [313,314]. These inconsistencies likely reflect the interaction of multiple factors, including fasting duration, circadian timing, habitual eating patterns, and individual susceptibility to hunger-related distraction or fatigue.

The clearest area of psychosocial concern relates to eating pathology. Here, the literature diverges sharply from the relatively neutral findings of metabolic RCTs. Observational and prospective studies consistently suggest that fasting used as a weight-control strategy is associated with greater ED psychopathology, including binge eating, bulimic symptoms, food preoccupation, and psychosocial impairment [85,315]. In adolescents and young adults, fasting behaviors have been shown to predict later binge eating and bulimic-spectrum pathology more strongly than more general measures of dietary restraint [83,316], suggesting that episodic restrictive practices may function as proximal behavioral risk factors rather than merely reflecting dieting intent. Importantly, this concern is especially relevant for females, who bear a greater overall burden of ED pathology, although population-based studies indicate that the association between IF and ED symptoms extends across genders, including males and gender-diverse individuals [317].

At the same time, the available evidence does not support the view that IF is intrinsically pathogenic in all contexts. Short-term experimental work suggests that a single prescribed fasting episode does not uniformly trigger binge eating or compensatory behaviors in all individuals [318]. Rather, fasting may act as a risk amplifier in those with pre-existing vulnerability traits such as disinhibition, impulsivity, shape/weight overvaluation, trauma exposure, or subclinical ED symptoms [85]. This interpretation is reinforced by adult clinical trials in supervised obesity treatment settings, where TRE or intermittent energy restriction has not consistently worsened disordered eating patterns or body image, and in some cases has even improved binge eating or uncontrolled eating relative to continuous daily restriction [87]. These findings suggest that the psychosocial impact of IF depends heavily on context: structured, clinically supervised, nutritionally adequate protocols in screened adult populations may have a relatively favorable safety profile, whereas unsupervised fasting adopted for weight or shape control in high-risk groups may increase psychological and behavioral harm.

From a sex-differential perspective, this issue is especially important because women and girls not only have higher baseline rates of EDs, but may also experience fasting in the context of menstrual-cycle-related appetite variability, body image pressures, and greater sensitivity to energy-related endocrine perturbations [319]. From a body-image perspective, emerging evidence suggests that IF may also be linked not only to ED symptomatology but to broader disturbances in body perception, including body dissatisfaction and dysmorphic traits, although the direction of this association appears context-dependent. Cross-sectional studies indicate that individuals engaging in IF tend to exhibit lower body image flexibility—a construct reflecting difficulty accepting body-related thoughts and feelings—which is itself strongly associated with body dissatisfaction and ED, with this association being more pronounced in women [320]. In parallel, fasting behaviors have been linked to higher levels of orthorexia, eating pathology, and body-dysmorphic symptomatology, particularly among adolescents and young adults [85,321]. However, contrasting evidence from adult weight-loss contexts suggests that structured IF may improve body image satisfaction and body appreciation, likely mediated by weight loss and perceived self-efficacy, highlighting a bidirectional relationship depending on population and motivation [322]. Importantly, sex differences appear to follow established gendered body ideals: in women, fasting-related body image disturbance tends to align with thinness-oriented dissatisfaction and heightened vulnerability to ED psychopathology, whereas in men, emerging evidence suggests a link with muscularity-oriented concerns). Experimental and clinical observations indicate that fasting may exacerbate body dissatisfaction in men and, in extreme cases, contribute to or intensify muscle dysmorphia symptomatology—characterized by a pathological perception of being insufficiently muscular—supporting the conceptualization of muscularity-oriented pathology as part of the ED spectrum [323,324].

Overall, the psychological effects of IF appear to be highly population-dependent. In structured adult metabolic trials, IF is generally associated with neutral psychosocial outcomes, with little evidence of meaningful deterioration in mood, anxiety, perceived stress, or quality of life. However, epidemiologic and prospective evidence indicates that fasting, when practiced as a self-directed weight-control behavior, is associated with higher ED psychopathology and related psychosocial impairment across genders, with particularly important implications for females and younger individuals. Thus, IF should not be viewed as either universally benign or inherently harmful from a psychological standpoint. Rather, its psychosocial safety likely depends on sex-linked vulnerability distributions, developmental stage, psychiatric history, and the degree of clinical supervision.

Future research should therefore move beyond treating psychosocial outcomes as secondary endpoints and should incorporate sex-stratified analyses, reproductive-state characterization, and more ecologically valid measurement approaches such as repeated daily ratings or ecological momentary assessment of hunger, craving, mood, sleep, and adherence. Longitudinal studies are especially needed to determine whether IF differentially influences the emergence or persistence of eating pathology, affective symptoms, and psychosocial functioning in women and men across different life stages. In particular, adolescents, individuals with prior EDs, and those with psychiatric comorbidity should be studied with explicit safety frameworks, as these groups are most likely to reveal clinically meaningful sex-dependent risks that remain obscured in current short-term adult trials.

## 5. Concluding Remarks and Integrative Perspective

The evidence reviewed in this work indicates that biological sex meaningfully modulates the metabolic, neuroendocrine, clinical, and behavioral responses to IF, although the strength of evidence varies substantially across contexts and remains insufficient for many direct male–female comparisons. At a general level, the literature supports the existence of sexually dimorphic responses to fasting in substrate metabolism, hormonal regulation, and tolerability, with women showing a greater reliance on lipid mobilization during fasting and a reproductive axis that is more tightly coupled to energy availability, while men appear to exhibit a more stable tolerance of moderate fasting regimens despite measurable endocrine adaptations, particularly in testosterone dynamics. These differences are unlikely to be merely quantitative and instead suggest that the optimal fasting “dose,” timing, and clinical applicability of IF may not be identical in women and men.

When examined in a more context-specific manner, these sex-related distinctions become especially relevant. In women of reproductive age, IF appears to require careful interpretation in light of menstrual status, energy availability, exercise load, and baseline metabolic phenotype. The available evidence suggests that moderate or well-designed TRE may be metabolically beneficial and even improve menstrual and ovulatory parameters in women with obesity or PCOS, whereas leaner, highly active, or otherwise vulnerable women may be more susceptible to LEA states, menstrual disturbances, and neuroendocrine dysregulation. During pregnancy and lactation, current data—derived mainly from Ramadan-type fasting—remain insufficient to support deliberate non-religious fasting for weight-loss purposes, and caution is warranted because maternal-fetal and maternal-infant metabolic demands are high. In postmenopausal women, by contrast, IF appears more promising as a strategy to improve body composition, insulin sensitivity, and selected cardiometabolic parameters, although menopause-specific long-term trials remain scarce. In men, the literature suggests that IF may be particularly useful as a cardiometabolic and body-composition strategy, especially in those with overweight or obesity, but its endocrine effects, including reductions in testosterone, should be interpreted in relation to leanness, physical activity, and overall energy balance rather than automatically viewed as pathological.

Across the comparative scenarios explored, the same overarching limitation repeatedly emerges: direct sex-comparative evidence is still remarkably limited. Whether considering exercise performance, chronic disease management, longevity-related mechanisms, or psychosocial outcomes, most studies either include mixed populations without adequate stratified analyses or are not powered to detect sex-by-intervention interactions. Consequently, many of the apparent differences discussed throughout this review are inferred from parallel male and female studies rather than demonstrated within the same experimental framework. This is particularly important because sex-specific effects may be masked when sex is treated merely as a descriptive covariate instead of a primary biological variable. The problem is not simply one of underrepresentation, but also of study design, since key modifiers such as menstrual phase, menopausal status, energy availability, training load, and baseline hormonal milieu are seldom incorporated systematically.

Therefore, the main conclusion of this review is not that IF should be considered broadly beneficial or harmful for one sex over the other, but rather that its effects are biologically and clinically context-dependent, and that sex is one of the different critical determinants of this variability. These findings support a more sex-informed and individualized interpretation of IF, in which biological sex is considered alongside age, reproductive stage, metabolic phenotype, body composition, lifestyle, and other sources of inter-individual heterogeneity. Future research should move beyond pooled analyses and explicitly address sex-specific responses through adequately powered trials, mechanistically informed study designs, and more detailed characterization of hormonal and physiological context. A summary of key research priorities is provided in Table 2. Only through such an approach will it be possible to define with greater precision when, for whom, and under what conditions intermittent fasting is safe, effective, and clinically meaningful.

## Figures and Tables

**Table 1:** Summary of potential benefits, reported risks or adverse effects, and population groups in which intermittent fasting (IF) may be more appropriate or require caution. LEA, low energy availability; TRE, time-restricted eating; ADF, alternate-day fasting; PF, periodic fasting; FMD, fasting-mimicking diet.

Population/Context	Potential Benefits	Reported Risks/Adverse Effects	Groups More Likely to Benefit	Groups Warranting Caution	Practical Considerations
Reproductive-age women	May improve insulin sensitivity, fasting glucose, waist circumference, triglycerides, and androgen-related markers in women with overweight/obesity, insulin resistance, metabolic syndrome, or PCOS.	Reduced leptin, altered GnRH/LH pulsatility, menstrual disturbances, fatigue, higher cortisol, reduced T3, and risk of LEA, especially under low energy reserves or high exercise load.	Women with overweight/obesity, insulin resistance, metabolic syndrome, or PCOS, particularly when energy intake remains adequate.	Lean women, women with low body fat, menstrual irregularities, high training loads, high psychosocial stress, sleep disruption, or history/risk of functional hypothalamic amenorrhea.	Prefer flexible or mild TRE; avoid aggressive ADF/PF when energy availability is low; monitor menstrual regularity, fatigue, hunger, training recovery, and cycle phase.
Women attempting conception/fertility treatment	Indirect potential benefits in PCOS through improved insulin resistance, hyperandrogenism, and menstrual cyclicity.	Uncertain effects on ovulation, luteal function, ovarian stimulation requirements, pregnancy complications, and IVF/ICSI outcomes; evidence remains indirect or observational.	Possibly women with PCOS-related metabolic dysfunction, under professional supervision.	Women with infertility, prior hypothalamic amenorrhea, low body weight, high exercise load, irregular cycles, or active fertility treatment.	IF should be individualized and considered experimental rather than evidence-based fertility care; prioritize stable weight, adequate micronutrient intake, ovulatory monitoring, and conservative TRE if used.
Pregnancy	Ramadan studies do not consistently show major adverse effects on birthweight or common obstetric outcomes, but evidence certainty is low. Some reports suggest reduced risk of gestational diabetes or excessive gestational weight gain in selected contexts.	Possible maternal hypoglycemia, ketonemia, metabolic stress, reduced placental weight, lower birthweight, reduced amniotic fluid index, altered fetal growth parameters, and unknown long-term offspring effects.	No clear group should be targeted for deliberate IF during pregnancy.	Pregnant women with diabetes, undernutrition, high workload, dehydration risk, sleep restriction, heat exposure, obstetric complications, or second/third-trimester prolonged fasting.	Deliberate non-religious IF should generally be discouraged; religious fasting should be individualized with medical counseling, hydration/nutrition planning, and risk stratification.
Lactation	Short-term religious fasting in healthy, well-nourished women appears to largely preserve milk macronutrient composition and short-term infant growth.	Possible micronutrient changes, maternal depletion, reduced milk quality/volume if intake is insufficient, especially with prolonged fasting or caloric restriction.	Healthy, well-nourished women undertaking short religious fasts with adequate intake during non-fasting hours.	Early postpartum women, undernourished women, high physical workload, prolonged fasting >20–24 h, or IF for weight loss.	Deliberate IF for weight loss is generally not recommended during breastfeeding; prioritize fluids, energy, micronutrients, and monitoring of milk supply and maternal symptoms.
Menopause/postmenopause	May improve body weight, fat mass, insulin resistance, blood pressure, lipid profile, oxidative stress, and inflammation; may be enhanced by resistance or combined exercise.	Evidence is limited by small trials and short duration; uncertainty remains regarding sarcopenia, visceral adiposity, long-term cardiometabolic outcomes, and menopause-specific endpoints.	Postmenopausal women with overweight/obesity, insulin resistance, cardiometabolic risk, or inflammatory/metabolic dysfunction.	Women at risk of sarcopenia, frailty, low protein intake, excessive caloric restriction, or poor training recovery.	Combine with resistance training when possible; ensure protein adequacy; avoid excessive restriction; monitor muscle mass, strength, cardiometabolic markers, and long-term adherence.
Men	May reduce fat mass, waist circumference, fasting insulin, blood pressure, and lipid-related cardiometabolic risk markers, particularly in men with overweight/obesity or visceral adiposity. When combined with resistance training and adequate intake, IF may reduce fat mass while generally preserving lean mass and strength.	Modest reductions in total/free testosterone have been reported, especially in lean, physically active men, usually without clear impairment in muscle mass or strength. Acute or prolonged fasting may increase cortisol and induce adaptive thyroid changes, while aggressive protocols combined with high training loads may increase fatigue, sleep disruption, under-fueling, impaired recovery, or reduced anabolic signaling. Evidence regarding male fertility remains preliminary and inconclusive.	Men with overweight/obesity, visceral adiposity, insulin resistance, or cardiometabolic risk; active men seeking body-composition improvements when energy and protein intake are adequate.	Lean young men, highly active men, athletes, men with high training loads, poor recovery, inadequate caloric/protein intake, or men actively attempting conception/subfertile men using aggressive IF without supervision.	IF should be viewed mainly as a cardiometabolic and body-composition strategy rather than a targeted endocrine or fertility intervention. Prioritize protein distribution, energy sufficiency, peri-training nutrition, sleep, recovery, and interpretation of testosterone changes in functional context
Male fertility/subfertility	Very preliminary data suggest possible favorable trends in sperm parameters after FMD in small studies; preclinical data suggest possible metabolic–fertility benefits.	Human evidence is limited and inconclusive; Ramadan studies report transient declines in progressive motility and semen volume.	No clear clinical target group yet; potentially men with metabolic dysfunction-related subfertility in research settings.	Men actively trying to conceive, subfertile men, or those with impaired sperm quality using aggressive IF without supervision.	IF should not be promoted as an evidence-based fertility intervention; future trials should assess sperm parameters, hormones, and live birth outcomes.
Older adults/cardiometabolic disease	Potential improvements in weight, insulin resistance, blood pressure, lipids, oxidative stress, inflammation, and metabolic flexibility.	Risk of inadequate intake, sarcopenia, frailty, medication-related hypoglycemia, dehydration, and poor tolerance if protocols are too restrictive.	Overweight/obese individuals with cardiometabolic risk, when clinically stable and nutritionally supervised.	Frail older adults, sarcopenic individuals, patients on glucose-lowering medication, or those with poor appetite/low protein intake.	Use conservative TRE rather than aggressive fasting; prioritize protein, resistance training, hydration, medication review, and clinical monitoring.

**Table 2:** Key research priorities for sex-specific investigation of intermittent fasting.

Domain	Research Priority	Rationale
Study design	Adequately powered sex-by-intervention randomized controlled trials	Most current studies are not designed to detect sex-specific effects, limiting causal inference
Sex-specific analysis	Sex-stratified outcomes as primary endpoints rather than covariates	Sex differences may be masked when not explicitly modeled
Female-specific design	Incorporation of menstrual cycle phase, contraceptive use, and menopausal status	Hormonal fluctuations significantly influence metabolic and neuroendocrine responses
Energy availability	Standardized assessment of energy intake, expenditure, and LEA	Many adverse outcomes may be driven by low energy availability rather than fasting per se
Exercise interaction	Controlled studies combining IF with resistance and endurance training across sexes	Current evidence is limited and often indirect, especially in women
Aging and longevity	Inclusion of validated biomarkers of biological aging (e.g., epigenetic clocks, telomeres, senescence markers)	Human data are currently limited to short-term metabolic endpoints
Reproductive outcomes	Assessment of ovulation, luteal function, fertility markers, and pregnancy outcomes	Evidence is indirect and largely extrapolated from metabolic or PCOS studies
Long-term outcomes	Longitudinal studies evaluating sustainability, safety, and clinical endpoints	Most trials are short-term and do not capture long-term adaptations or risks
Body composition and bone health	Evaluation of lean mass, sarcopenia risk, and bone mineral density	Particularly relevant in women, older adults, and under energy restriction
Population diversity	Inclusion of diverse populations (age, BMI, training status, metabolic phenotype)	Responses to IF are highly context-dependent
Behavioral and adherence factors	Assessment of real-world adherence, hunger, fatigue, and psychosocial responses	Gender-related factors may influence implementation and outcomes

## Data Availability

No new data were created or analyzed in this study. Data sharing is not applicable to this article.

## Abbreviations

The following abbreviations are used in this manuscript:

ADF	alternate-day fasting;
AMPK	AMP-activated protein kinase;
BDNF	brain-derived neurotrophic factor;
BMAL1	brain and muscle ARNT-like 1;
CLOCK	circadian locomotor output cycles kaput;
CRP	C-reactive protein;
DHEA	dehydroepiandrosterone;
FMD	fasting-mimicking diet;
FFA	free fatty acids;
FOXO	forkhead box O transcription factors;
FSH	follicle-stimulating hormone;
GnRH	gonadotropin-releasing hormone;
HDAC	histone deacetylase;
HPA axis	hypothalamic–pituitary–adrenal axis;
HPG axis	hypothalamic–pituitary–gonadal axis;
IF	intermittent fasting;
IGF-1	insulin-like growth factor 1;
IL-6	interleukin 6;
LC3	microtubule-associated protein 1 light chain 3;
LEA	low energy availability;
LH	luteinizing hormone;
mTORC1	mechanistic target of rapamycin complex 1;
NAD^+^	nicotinamide adenine dinucleotide (oxidized form);
NADH	nicotinamide adenine dinucleotide (reduced form);
NLRP3	NOD-, LRR- and pyrin domain-containing protein 3;
NRF2	nuclear factor erythroid 2–related factor 2;
PCOS	polycystic ovary syndrome;
PF	periodic fasting;
PGC-1α	peroxisome proliferator-activated receptor gamma coactivator 1-alpha;
rT3	reverse triiodothyronine;
SCFAs	short-chain fatty acids;
SHBG	sex hormone-binding globulin;
SIRT	sirtuin;
T3	triiodothyronine;
T4	thyroxine;
TRE	time-restricted eating;
TNF-α	tumor necrosis factor alpha;
TRH	thyrotropin-releasing hormone;
TSH	thyroid-stimulating hormone;
ULK1	unc-51 like autophagy activating kinase 1.
